# From tuberculosis bedside to bench: UBE2B splicing as a potential biomarker and its regulatory mechanism

**DOI:** 10.1038/s41392-023-01346-2

**Published:** 2023-02-24

**Authors:** Mengyuan Lyu, Jian Zhou, Yanbing Zhou, Weelic Chong, Wei Xu, Hongli Lai, Lu Niu, Yang Hai, Xiaojun Yao, Sheng Gong, Qinglan Wang, Yi Chen, Yili Wang, Liyu Chen, Zengwanggema, Jiongjiong Zeng, Chengdi Wang, Binwu Ying

**Affiliations:** 1 Department of Laboratory Medicine, West China Hospital, Sichuan University, Chengdu, Sichuan 610041 China; 2 Department of Thoracic Surgery, West China Hospital, Sichuan University, Chengdu, Sichuan 610041 China; 3 Sidney Kimmel Medical College, Thomas Jefferson University, Philadelphia, PA 19107 USA; 4 Department of Biostatistics, Princess Margaret Cancer Centre, University Health Network, Toronto, Ontario, M5G 1L7 Canada; 5 Dalla Lana School of Public Health, University of Toronto, Toronto, ON M5T 3M7 Canada; 6 Institute of Thoracic Oncology, West China Hospital, Sichuan University, Chengdu, Sichuan 610041 China; 7 Department of Radiology, Thomas Jefferson University Hospital, Philadelphia, PA 19107 USA; 8 Department of Thoracic Surgery, The Public and Health Clinic Centre of Chengdu, Chengdu, Sichuan 610066 China; 9 Department of Respiratory and Critical Care Medicine, Frontiers Science Center for Disease-related Molecular Network, West China Hospital, Sichuan University, Chengdu, Sichuan 610213 China; 10 Department of Infectious Diseases, West China Hospital, Sichuan University, Chengdu, Sichuan 610041 China; 11 Zhaojue People’s Hospital of Liangshan Prefecture, Liangshan Prefecture, Sichuan 616150 China; 12 Department of Laboratory Medicine, Ganzi People’s Hospital, Ganzi Prefecture, Sichuan 626099 China

**Keywords:** Infectious diseases, Biomarkers

## Abstract

Alternative splicing (AS) is an important approach for pathogens and hosts to remodel transcriptome. However, tuberculosis (TB)-related AS has not been sufficiently explored. Here we presented the first landscape of TB-related AS by long-read sequencing, and screened four AS events (S100A8-intron1-retention intron, RPS20-exon1-alternaitve promoter, KIF13B-exon4-skipping exon (SE) and UBE2B-exon7-SE) as potential biomarkers in an in-house cohort-1. The validations in an in-house cohort-2 (2274 samples) and public datasets (1557 samples) indicated that the latter three AS events are potential promising biomarkers for TB diagnosis, but not for TB progression and prognosis. The excellent performance of classifiers further underscored the diagnostic value of these three biomarkers. Subgroup analyses indicated that UBE2B-exon7-SE splicing was not affected by confounding factors and thus had relatively stable performance. The splicing of UBE2B-exon7-SE can be changed by heat-killed *mycobacterium tuberculosis* through inhibiting SRSF1 expression. After heat-killed *mycobacterium tuberculosis* stimulation, 231 ubiquitination proteins in macrophages were differentially expressed, and most of them are apoptosis-related proteins. Taken together, we depicted a global TB-associated splicing profile, developed TB-related AS biomarkers, demonstrated an optimal application scope of target biomarkers and preliminarily elucidated *mycobacterium tuberculosis*-host interaction from the perspective of splicing, offering a novel insight into the pathophysiology of TB.

## Introduction

Tuberculosis (TB) remains one of the deadliest infectious diseases.^1^ Early diagnosis and timely treatment are critical to controlling TB.^2^ Laboratory detections (etiological tests and immunological tests) serve as the main approaches for TB diagnosis and prognosis monitoring. Etiological tests (sputum smear, culture, etc.) provide direct evidence, but they have disadvantages of insufficient accuracy, and inapplicability to negative-bacteria cases.^3^ Although immunological tests (interferon gamma release assay, T-SPOT^®^.TB test, etc.) offer indirect evidence, they have a relatively wide application scope and can be applied to negative-bacteria cases. In essence, immunological tests are a kind of biomarker-driven strategy, and the characteristics of biomarkers largely determine the performance of such detections.^4–6^ Therefore, exploring various promising biomarkers has always been the focus in the TB filed. Of these, transcriptomic molecules are receiving attention due to low detection cost, good repeatability and excellent performance.^7–9^

Typically, these transcriptome biomarkers were developed through gene expression-based screening strategy. In this strategy, a gene is seen as a unit and the inherent heterogeneity of a gene itself is ignored, leading to a biased capture of biomarkers/targets.^10^ Alternative splicing (AS) allows researchers to interpretate the transcriptome at a finer level. AS refers to the process by which a pre-mRNA is spliced by different ways into mature mRNAs with different exons or introns.^11^ AS occurs on more than 95% of human genes and contributes greatly to the transcriptome and proteome richness.^11^ AS reflects changes in the transcript expression, the proportion between transcripts, as well as changes in the function or structure of corresponding proteins. Multiple observation perspectives of AS confer its ability to capture disease related-tiny signals, and ensure its sensitivity and specificity as biomarkers.

AS is one of the major approaches that hosts and pathogens remodel the transcriptome.^12,13^ In TB, many studies reported the roles of AS events (RAB8B splicing, IL-12Rβ1 splicing, etc.) in the *mycobacterium tuberculosis* (MTB)-host interactions,^14–18^ highlighting the importance of AS in TB development. Further exploring the potential of AS events as biomarkers and the regulatory mechanism of these biomarkers in TB is necessary for a deeper understanding of TB. Serine/arginine rich splicing factor (SRSF) family widely participates in the pathogen-host interactions by regulating the splicing of immune-related genes, and this family is able to respond to external stimulations.^19^ These characteristics indicate that SRSF family might be a target for MTB to regulate host splicing.

Here, we identified AS biomarkers in 2 in-house cohorts and 7 public datasets, and further investigated the regulatory mechanism of these biomarkers in TB. We conducted Oxford Nanopore Technologies (ONT) sequencing in the in-house cohort-1 to select biomarkers. We validated the selected biomarkers in an independent in-house cohort-2 (2274 participants) and 7 public datasets (1557 samples). Furthermore, we explored the regulatory mechanism of MTB on the target AS biomarker through SRSF in cell models. We aimed to offer outperforming TB-related AS biomarkers, clarify the optimal application scopes of these biomarkers and preliminarily uncover the regulatory mechanism and biological roles of the target AS event.

## Results

### TB-related AS profiles and biomarker selection

**Fig. 1:**
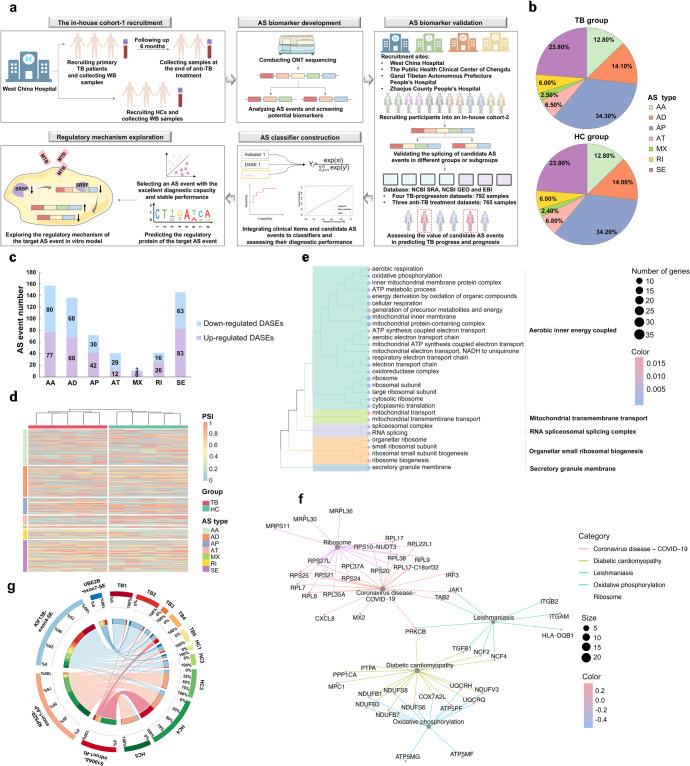
The splicing profiling of primary TB patients and HCs in an in-house cohort-1 based on ONT sequencing. **a** The flowchart of the study design. **b** Pie chart showing the proportion of alternative splicing types in different groups. **c** Stacked bar plots of differential alternative splicing events. **d** Heatmaps showing the PSI values of differential alternative splicing events. **e** GO enrichment analysis of DASE-related genes. **f** KEGG enrichment analysis of DASE-related genes. **g** Circus plots showing the splicing of selected four events in different samples. The left inner circle exhibited the proportion of PSI values of different samples in the accordingly splicing events. The right inner circle exhibited the proportion of PSI values of different splicing events in the accordingly samples. The line thickness inside the circle indicated the proportion described above. TB tuberculosis, WB whole blood, HC healthy control, AS alternative splicing, ONT Oxford Nanopore Technologies, SRSF serine/arginine-rich splicing factor, MTB *mycobacterium tuberculosis*, DASE differential alternative splicing event; PSI percent spliced in, AA alternate acceptor site, AD alternate donor site, AP alternate promoter, AT alternate terminator, SE skipping exon, ME mutually exclusive exons, RI intron retention, S100A8 S100 Calcium Binding Protein A8, RPS20 Ribosomal Protein S20, KIF13B Kinesin Family Member 13B, UBE2B Ubiquitin Conjugating Enzyme E2 B

We first performed ONT sequencing in the in-house cohort-1 comprising five primary TB patients and five healthy controls (HCs) (Fig. 1a, Supplementary Table S1 and Supplementary Fig. S1). ONT sequencing detected 72,007 and 75,443 AS events in the TB and HC groups, respectively. In both groups, alternative promoter (AP) events accounted for most of the events, followed by skipping exon (SE) events (Fig. 1b). And there was no difference in the proportion of AS types between the TB and HC groups. Totally, 605 events were differentially spliced between the TB and HC groups (Fig. 1c, d). These differential AS events (DASEs)-related genes were enriched in the pathways of oxidative phosphorylation and electron transport chain (Fig. 1e, f).

For these DASEs, the expression of involved transcripts (transcripts per million>1), average percent spliced in (PSI) within a group (PSI > 0.01) and potential biological roles were further considered as the screening criteria. We then identified four events that harboring the potential as TB diagnostic biomarkers (Table 1). TB patients exhibited lower PSI values of both S100 Calcium Binding Protein A8 (S100A8)-intron1-retention intron (delta PSI (ΔPSI) = −0.14, *P* = 0.031) and Ribosomal Protein S20 (RPS20)-exon1-AP (ΔPSI = −0.56, *P* = 0.022) than HCs. Compared with the HC group, the PSI values of Kinesin Family Member 13B (KIF13B)-exon4-SE (ΔPSI = 0.27, *P* = 0.034) and Ubiquitin Conjugating Enzyme E2 B (UBE2B)-exon7-SE (ΔPSI = 0.05, *P* = 0.047) in the TB group were increased (Fig. 1g).

**Table 1:** The detailed information of selected 4 alternative splicing events^a^

Gene	Splicing type	Chromosome	Chain	Splicing site
S100A8	RI	1	-	153390395:153390557–153391041:153391073
RPS20	AP	8	-	56074159–56074254:56074356:56074159–56074381:56074510
KIF13B	SE	8	-	29191057–29196187; 29196199–29245346
UBE2B	SE	5	+	134388413–134388977; 134389052–134390225

^a^The spliced exons or introns (e.g. exon7) in this work were named according to the order of all exons or introns that appeared in all transcripts of the corresponding gene. *S100A8* S100 Calcium Binding Protein A8, *RI* retention intron, *RPS20* Ribosomal Protein S20, *AP* alternative promoter, *KIF13B* Kinesin Family Member 13B, *SE* skipping exon, *UBE2B* Ubiquitin Conjugating Enzyme E2 B

The splicing of these 4 events in TB patients with different outcomes was also analyzed to preliminarily explore their potential to predict TB prognosis (Supplementary Table S2 and Supplementary Fig. S2). The statistics of ONT sequencing is shown in Supplementary Table S3.

### TB-associated AS biomarker validation

**Fig. 2:**
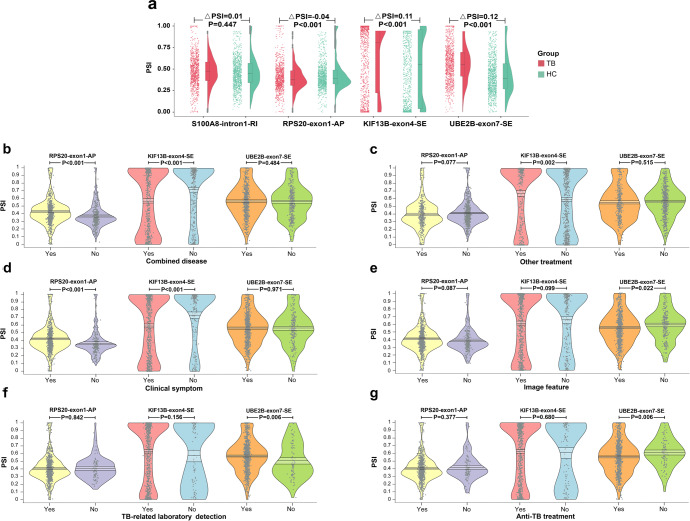
The splicing of selected four events in the in-house cohort-2. **a** The splicing of selected four events in the in-house cohort-2. The left scatters and right violin plots showed the distribution of PSI values and data density, respectively. The middle boxplot showed the median, two hinges, two whiskers, and outlying points. **b**–**g** Pirate plots of the target three splicing events in subgroups divided by combined disease, other treatment, clinical symptom, image feature, TB-related laboratory detection and anti-TB treatment, respectively. Points represented the raw data. The vertical bar and bands showed the mean and 95% highest density interval, respectively. The bean exhibited the data density. PSI percent spliced in, TB tuberculosis, HC healthy control, S100A8 S100 Calcium Binding Protein A8, RI intron retention, RPS20 Ribosomal Protein S20, AP alternate promoter, KIF13B Kinesin Family Member 13B, SE skipping exon, UBE2B Ubiquitin Conjugating Enzyme E2 B

Altogether 2274 participants comprising of 1036 TB patients and 1238 HCs were included in our in-house cohort-2 (Supplementary Fig. S1 and Supplementary Table S4). Significant splicing differences of RPS20-exon1-AP, KIF13B-exon4-SE and UBE2B-exon7-SE between the TB and HC groups were still observed (ΔPSI = −0.04, *P* < 0.001; ΔPSI = 0.11, *P* < 0.001; ΔPSI = 0.12, *P* < 0.001). However, no splicing difference of S100A8-intron1-retention intron was found between the two groups (ΔPSI = 0.01, *P* = 0.447) (Fig. 2a), and thus S100A8-intron1-retention intron was further excluded from subsequent analysis.

### Relationship between AS biomarkers and clinical characteristics

Based on the in-house cohort-2, we analyzed the splicing differences of target three AS events between subgroups that were stratified by clinical characteristics. For TB patients, no significant splicing differences of UBE2B-exon7-SE were found between subgroups that were divided by sex (*P* = 0.447), smoking history (*P* = 0.175), or drinking history (*P* = 0.673). No significant splicing differences of UBE2B-exon7-SE were found between the patients with and without combined disease (*P* = 0.484), as well as between the patients with and without or other treatment (hypotensive treatment, hypoglycemic treatment, etc.) (*P* = 0.515) (Fig. 2b, c). Although UBE2B-exon7-SE splicing did not significantly differ between the patients with and without clinical symptoms (*P* = 0.971) (Fig. 2d), it observably differed between the patients with and without image features (*P* = 0.022) (Fig. 2e). Additionally, the PSI value of UBE2B-exon7-SE significantly increased in the patients with positive results of TB-related laboratory detections (*P* = 0.006) (Fig. 2f). The PSI value of UBE2B-exon7-SE were reduced in the anti-TB treatment subgroup compared to the non-treatment subgroup (*P* = 0.006) (Fig. 2g). The detailed results of subgroup analyses are provided in Table 2.

**Table 2:** The results of subgroup analyses

		RPS20-exon1-AP			KIF13B-exon4-SE			UBE2B-exon7-SE		
		HC	TB	*P*	HC	TB	*P*	HC	TB	*P*
**Demographic characteristics**										
Sex	Female	0.46 (0.21)	0.41 (0.17)	0.001	0.50 (0.41)	0.60 (0.39)	<0.001	0.45 (0.25)	0.57 (0.21)	<0.001
	Male	0.43 (0.18)	0.40 (0.17)	0.003	0.52 (0.41)	0.64 (0.39)	<0.001	0.43 (0.23)	0.56 (0.21)	<0.001
	*P*	0.028	0.211	-	0.418	0.131	-	0.170	0.447	-
Ethnicity	Han	0.42 (0.20)	0.36 (0.14)	<0.001	0.54 (0.40)	0.73 (0.34)	<0.001	0.43 (0.24)	0.55 (0.21)	<0.001
	Tibet	0.50 (0.19)	0.45 (0.17)	<0.001	0.42 (0.42)	0.51 (0.39)	<0.001	0.44 (0.23)	0.55 (0.21)	<0.001
	Yi	0.51 (0.10)	0.47 (0.22)	0.146	0.54 (0.36)	0.49 (0.42)	0.345	0.52 (0.22)	0.68 (0.20)	<0.001
	Others	-	0.36 (0.08)	-	-	0.74 (0.40)	-	-	0.49 (0.18)	-
	*P*	<0.001	<0.001		<0.001	<0.001		0.004	<0.001	
**Personal history**										
Smoking history	No	-	0.39 (0.16)	-	-	0.67 (0.37)	-	-	0.55 (0.21)	-
	Yes	-	0.43 (0.18)	-	-	0.56 (0.40)	-	-	0.57 (0.21)	-
	*P*	-	0.001	-	-	<0.001	-	-	0.175	-
Drinking history	No	-	0.38 (0.16)	-	-	0.67 (0.37)	-	-	0.56 (0.21)	-
	Yes	-	0.44 (0.17)	-	-	0.57 (0.40)	-	-	0.57 (0.20)	-
	*P*	-	<0.001	-	-	<0.001	-	-	0.673	-
**Combined disease**										
Combined disease	No	0.44 (0.20)	0.37 (0.15)	<0.001	0.51 (0.41)	0.72 (0.36)	<0.001	0.44 (0.24)	0.56 (0.21)	<0.001
	Yes	-	0.43 (0.18)	-	-	0.57 (0.39)	-	-	0.57 (0.21)	-
	*P*	-	<0.001	-	-	<0.001	-	-	0.484	-
**Detailed combined diseases**										
Syphilis	No	0.44 (0.20)	0.40 (0.17)	<0.001	0.51 (0.41)	0.64 (0.39)	<0.001	0.44 (0.24)	0.56 (0.21)	<0.001
	Yes	-	0.45 (0.18)	-	-	0.56 (0.38)		-	0.58 (0.20)	-
	*P*	-	<0.001	-	-	0.028	-	-	0.316	-
AIDS	No	0.44 (0.20)	0.40 (0.17)	<0.001	0.51 (0.41)	0.63 (0.39)	<0.001	0.44 (0.24)	0.55 (0.22)	<0.001
	Yes	-	0.43 (0.16)	-	-	0.60 (0.36)		-	0.58 (0.19)	-
	*P*	-	0.038	-	-	0.310	-	-	0.050	-
Parasite infection	No	0.44 (0.20)	0.39 (0.16)	<0.001	0.51 (0.41)	0.65 (0.39)	<0.001	0.44 (0.24)	0.54 (0.22)	<0.001
	Yes	-	0.44 (0.17)	-	-	0.57 (0.38)	-	-	0.61 (0.20)	-
	*P*	-	<0.001	-	-	0.001	-	-	<0.001	-
Unknown infectious disease	No	0.44 (0.20)	0.38 (0.16)	<0.001	0.51 (0.41)	0.69 (0.37)	<0.001	0.44 (0.24)	0.55 (0.22)	<0.001
	Yes	-	0.44 (0.17)	-	-	0.53 (0.39)	-	-	0.58 (0.20)	-
	*P*	-	<0.001	-	-	<0.001	-	-	0.014	-
Hepatitis	No	0.44 (0.20)	0.40 (0.17)	<0.001	0.51 (0.41)	0.63 (0.39)	<0.001	0.44 (0.24)	0.55 (0.22)	<0.001
	Yes	-	0.41 (0.17)	-	-	0.61 (0.37)	-	-	0.59 (0.20)	-
	*P*	-	0.609	-	-	0.311	-	-	0.008	-
Other hepatic disease	No	0.44 (0.20)	0.39 (0.16)	<0.001	0.51 (0.41)	0.65 (0.39)	<0.001	0.44 (0.24)	0.54 (0.22)	<0.001
	Yes	-	0.43 (0.18)	-	-	0.58 (0.38)	-	-	0.60 (0.20)	-
	*P*	-	<0.001	-	-	0.008	-	-	<0.001	-
Psychological disorder	No	0.44 (0.20)	0.40 (0.16)	<0.001	0.51 (0.41)	0.64 (0.39)	<0.001	0.44 (0.24)	0.54 (0.22)	<0.001
	Yes	-	0.43 (0.17)	-	-	0.58 (0.38)	-	-	0.61 (0.19)	-
	*P*	-	0.003	-	-	0.021	-	-	<0.001	-
Sleep disorder	No	0.44 (0.20)	0.39 (0.16)	<0.001	0.51 (0.41)	0.65 (0.39)	<0.001	0.44 (0.24)	0.54 (0.22)	<0.001
	Yes	-	0.43 (0.17)	-	-	0.58 (0.38)	-	-	0.61 (0.19)	-
	*P*	-	<0.001	-	-	0.007	-	-	<0.001	-
Cerebral disease	No	0.44 (0.20)	0.39 (0.16)	<0.001	0.51 (0.41)	0.65 (0.38)	<0.001	0.44 (0.24)	0.54 (0.22)	<0.001
	Yes	-	0.44 (0.18)	-	-	0.57 (0.39)	-	-	0.61 (0.19)	-
	*P*	-	<0.001	-	-	0.003	-	-	<0.001	-
Cardiac disease	No	0.44 (0.20)	0.39 (0.16)	<0.001	0.51 (0.41)	0.67 (0.38)	<0.001	0.44 (0.24)	0.55 (0.22)	<0.001
	Yes	-	0.44 (0.18)	-	-	0.55 (0.38)	-	-	0.59 (0.20)	-
	*P*	-	<0.001	-	-	<0.001	-	-	0.007	-
Renal disease	No	0.44 (0.20)	0.39 (0.16)	<0.001	0.51 (0.41)	0.65 (0.39)	<0.001	0.44 (0.24)	0.54 (0.22)	<0.001
	Yes	-	0.43 (0.17)	-	-	0.58 (0.38)	-	-	0.61 (0.20)	-
	*P*	-	<0.001	-	-	0.016	-	-	<0.001	-
Cancer	No	0.44 (0.20)	0.39 (0.16)	<0.001	0.51 (0.41)	0.64 (0.39)	<0.001	0.44 (0.24)	0.54 (0.22)	<0.001
	Yes	-	0.43 (0.17)	-	-	0.59 (0.38)	-	-	0.61 (0.19)	-
	*P*	-	0.001	-	-	0.033	-	-	<0.001	-
Diabetes mellitus	No	0.44 (0.20)	0.39 (0.16)	<0.001	0.51 (0.41)	0.65 (0.38)	<0.001	0.44 (0.24)	0.54 (0.21)	<0.001
	Yes	-	0.43 (0.17)	-	-	0.58 (0.39)	-	-	0.61 (0.20)	-
	*P*	-	<0.001	-	-	0.007	-	-	<0.001	-
Electrolytes disorder	No	0.44 (0.20)	0.39 (0.16)	<0.001	0.51 (0.41)	0.66 (0.38)	<0.001	0.44 (0.24)	0.54 (0.22)	<0.001
	Yes	-	0.44 (0.18)	-	-	0.56 (0.39)	-	-	0.60 (0.19)	-
	*P*	-	<0.001	-	-	<0.001	-	-	<0.001	-
Anemia	No	0.44 (0.20)	0.39 (0.16)	<0.001	0.51 (0.41)	0.66 (0.38)	<0.001	0.44 (0.24)	0.54 (0.21)	<0.001
	Yes	-	0.43 (0.18)	-	-	0.56 (0.38)	-	-	0.60 (0.20)	-
	*P*	-	<0.001	-	-	<0.001	-	-	<0.001	-
Hypertension	No	0.44 (0.20)	0.40 (0.17)	<0.001	0.51 (0.41)	0.64 (0.39)	<0.001	0.44 (0.24)	0.54 (0.22)	<0.001
	Yes	-	0.43 (0.17)	-	-	0.59 (0.38)	-	-	0.61 (0.20)	-
	*P*	-	0.003	-	-	0.050	-	-	<0.001	-
Hyperlipemia	No	0.44 (0.20)	0.39 (0.16)	<0.001	0.51 (0.41)	0.66 (0.38)	<0.001	0.44 (0.24)	0.54 (0.22)	<0.001
	Yes	-	0.44 (0.18)	-	-	0.55 (0.39)	-	-	0.60 (0.19)	-
	*P*	-	<0.001	-	-	<0.001	-	-	<0.001	-
Pulmonary hypertension	No	0.44 (0.20)	0.39 (0.16)	<0.001	0.51 (0.41)	0.66 (0.38)	<0.001	0.44 (0.24)	0.54 (0.22)	<0.001
	Yes	-	0.44 (0.18)	-	-	0.56 (0.39)	-	-	0.60 (0.19)	-
	*P*	-	<0.001	-	-	<0.001	-	-	<0.001	-
Hypoproteinemia	No	0.44 (0.20)	0.39 (0.16)	<0.001	0.51 (0.41)	0.67 (0.38)	<0.001	0.44 (0.24)	0.55 (0.22)	<0.001
	Yes	-	0.43 (0.17)	-	-	0.56 (0.38)	-	-	0.59 (0.20)	-
	*P*	-	<0.001	-	-	<0.001	-	-	0.001	-
**Other treatment**										
Other treatment	No	0.44 (0.20)	0.41 (0.16)	0.002	0.51 (0.41)	0.59 (0.38)	<0.001	0.44 (0.24)	0.57 (0.20)	<0.001
	Yes	-	0.39 (0.18)	-	-	0.67 (0.38)	-	-	0.56 (0.23)	-
	*P*	-	0.077	-	-	0.002	-	-	0.515	-
**Detailed other drugs’ usage**										
Hypotensive drugs	No	0.44 (0.20)	0.41 (0.16)	<0.001	0.51 (0.41)	0.61 (0.38)	<0.001	0.44 (0.24)	0.56 (0.21)	<0.001
	Yes	-	0.39 (0.19)	-	-	0.67 (0.39)	-	-	0.58 (0.22)	-
	*P*	-	0.206	-	-	0.054	-	-	0.105	-
Hypoglycemic drugs	No	0.44 (0.20)	0.41 (0.16)	<0.001	0.51 (0.41)	0.62 (0.38)	<0.001	0.44 (0.24)	0.55 (0.21)	<0.001
	Yes	-	0.40 (0.19)	-	-	0.65 (0.40)	-	-	0.59 (0.22)	-
	*P*	-	0.850	-	-	0.317	-	-	0.023	-
Prednisone	No	0.44 (0.20)	0.41 (0.16)	<0.001	0.51 (0.41)	0.61 (0.39)	<0.001	0.44 (0.24)	0.56 (0.21)	<0.001
	Yes	-	0.39 (0.18)	-	-	0.68 (0.38)	-	-	0.56 (0.22)	-
	*P*	-	0.138	-	-	0.010	-	-	0.880	-
Hepatic protector	No	0.44 (0.20)	0.41 (0.16)	<0.001	0.51 (0.41)	0.60 (0.39)	<0.001	0.44 (0.24)	0.56 (0.20)	<0.001
	Yes	-	0.39 (0.18)	-	-	0.67 (0.38)	-	-	0.56 (0.23)	-
	*P*	-	0.114	-	-	0.013	-	-	0.864	-
**Clinical symptom**										
Clinical symptom	No	0.44 (0.20)	0.36 (0.15)	<0.001	0.51 (0.41)	0.73 (0.36)	<0.001	0.44 (0.24)	0.56 (0.22)	<0.001
	Yes	-	0.42 (0.17)	-	-	0.59 (0.39)	-	-	0.56 (0.21)	-
	*P*	-	<0.001	-	-	<0.001	-	-	0.971	-
**Detailed clinical symptoms**										
Cough	No	0.44 (0.20)	0.38 (0.15)	<0.001	0.51 (0.41)	0.69 (0.37)	<0.001	0.44 (0.24)	0.56 (0.21)	<0.001
	Yes	-	0.44 (0.18)	-	-	0.56 (0.39)	-	-	0.56 (0.21)	-
	*P*	-	<0.001	-	-	<0.001	-	-	0.673	-
Chest pain	No	0.44 (0.20)	0.40 (0.16)	<0.001	0.51 (0.41)	0.64 (0.38)	<0.001	0.44 (0.24)	0.55 (0.21)	<0.001
	Yes	-	0.42 (0.18)	-	-	0.56 (0.40)	-	-	0.60 (0.21)	-
	*P*	-	0.295	-	-	0.006	-	-	0.005	-
Fatigue	No	0.44 (0.20)	0.39 (0.16)	<0.001	0.51 (0.41)	0.67 (0.37)	<0.001	0.44 (0.24)	0.55 (0.21)	<0.001
	Yes	-	0.45 (0.18)	-	-	0.52 (0.40)	-	-	0.59 (0.21)	-
	*P*	-	<0.001	-	-	<0.001	-	-	0.004	-
Fever	No	0.44 (0.20)	0.40 (0.16)	<0.001	0.51 (0.41)	0.63 (0.39)	<0.001	0.44 (0.24)	0.56 (0.21)	<0.001
	Yes	-	0.42 (0.17)	-	-	0.61 (0.39)	-	-	0.56 (0.22)	-
	*P*	-	0.038	-	-	0.453	-	-	0.900	-
Hemoptysis	No	0.44 (0.20)	0.40 (0.17)	<0.001	0.51 (0.41)	0.65 (0.38)	<0.001	0.44 (0.24)	0.56 (0.21)	<0.001
	Yes	-	0.42 (0.17)	-	-	0.58 (0.39)	-	-	0.62 (0.18)	-
	*P*	-	0.040	-	-	0.006	-	-	<0.001	-
Night sweat	No	0.44 (0.20)	0.39 (0.16)	<0.001	0.51 (0.41)	0.67 (0.37)	<0.001	0.44 (0.24)	0.54 (0.21)	<0.001
	Yes	-	0.43 (0.17)	-	-	0.55 (0.39)	-	-	0.60 (0.21)	-
	*P*	-	0.003	-	-	<0.001	-	-	<0.001	-
**Image feature**										
Image feature	No	0.44 (0.20)	0.39 (0.16)	<0.001	0.51 (0.41)	0.66 (0.38)	<0.001	0.44 (0.24)	0.59 (0.20)	<0.001
	Yes	-	0.41 (0.17)	-	-	0.61 (0.39)	-	-	0.55 (0.21)	-
	*P*	-	0.087	-	-	0.099	-	-	0.022	-
**Detailed image features**										
Pulmonary cavity	No	0.44 (0.20)	0.39 (0.16)	<0.001	0.51 (0.41)	0.66 (0.38)	<0.001	0.44 (0.24)	0.56 (0.21)	<0.001
	Yes	-	0.45 (0.19)	-	-	0.51 (0.40)	-	-	0.57 (0.22)	-
	*P*	-	<0.001	-	-	<0.001	-	-	0.622	-
Pulmonary calcification	No	0.44 (0.20)	0.39 (0.16)	<0.001	0.51 (0.41)	0.66 (0.38)	<0.001	0.44 (0.24)	0.56 (0.21)	<0.001
	Yes	-	0.44 (0.17)	-	-	0.57 (0.39)	-	-	0.56 (0.21)	-
	*P*	-	<0.001	-	-	<0.001	-	-	0.718	-
Miliary mottling	No	0.44 (0.20)	0.41 (0.17)	<0.001	0.51 (0.41)	0.62 (0.39)	<0.001	0.44 (0.24)	0.56 (0.21)	<0.001
	Yes	-	0.38 (0.10)	-	-	0.82 (0.31)	-	-	0.60 (0.20)	-
	*P*	-	0.348	-	-	0.001	-	-	0.233	-
Pulmonary nodule	No	0.44 (0.20)	0.42 (0.17)	0.019	0.51 (0.41)	0.58 (0.39)	0.001	0.44 (0.24)	0.57 (0.21)	<0.001
	Yes	-	0.38 (0.16)	-	-	0.71 (0.36)	-	-	0.54 (0.21)	-
	*P*	-	<0.001	-	-	<0.001	-	-	0.022	-
Pneumonedema	No	0.44 (0.20)	0.39 (0.17)	<0.001	0.51 (0.41)	0.66 (0.37)	<0.001	0.44 (0.24)	0.55 (0.21)	<0.001
	Yes	-	0.43 (0.17)	-	-	0.56 (0.40)	-	-	0.58 (0.21)	-
	*P*	-	0.001	-	-	<0.001	-	-	0.090	-
Lymphadenopathy	No	0.44 (0.20)	0.38 (0.15)	<0.001	0.51 (0.41)	0.70 (0.36)	<0.001	0.44 (0.24)	0.56 (0.21)	<0.001
	Yes	-	0.46 (0.18)	-	-	0.50 (0.39)	-	-	0.57 (0.21)	-
	*P*	-	<0.001	-	-	<0.001	-	-	0.524	-
**TB-related laboratory detection**										
TB-related detection	No	0.44 (0.20)	0.41 (0.15)	0.111	0.51 (0.41)	0.57 (0.39)	0.173	0.44 (0.24)	0.51 (0.22)	0.006
	Yes	-	0.41 (0.17)	-	-	0.63 (0.39)	-	-	0.57 (0.21)	-
	*P*	-	0.842	-	-	0.156	-	-	0.006	-
**Detailed TB-related laboratory detections**										
Smear	No	0.44 (0.20)	0.41 (0.17)	<0.001	0.51 (0.41)	0.62 (0.39)	<0.001	0.44 (0.24)	0.55 (0.21)	<0.001
	Yes	-	0.41 (0.16)	-	-	0.65 (0.38)	-	-	0.60 (0.19)	-
	*P*	-	0.994	-	-	0.333	-	-	0.010	-
IGRA	No	0.44 (0.20)	0.42 (0.17)	0.079	0.51 (0.41)	0.56 (0.41)	0.045	0.44 (0.24)	0.56 (0.22)	<0.001
	Yes	-	0.39 (0.16)	-	-	0.67 (0.36)	-	-	0.56 (0.21)	-
	*P*	-	0.006	-	-	<0.001	-	-	0.911	-
Culture	No	0.44 (0.20)	0.40 (0.16)	<0.001	0.51 (0.41)	0.64 (0.38)	<0.001	0.44 (0.24)	0.54 (0.21)	<0.001
	Yes	-	0.41 (0.18)	-	-	0.59 (0.40)	-	-	0.61 (0.20)	-
	*P*	-	0.488	-	-	0.027	-	-	<0.001	-
TB-DNA	No	0.44 (0.20)	0.40 (0.17)	<0.001	0.51 (0.41)	0.65 (0.38)	<0.001	0.44 (0.24)	0.55 (0.22)	<0.001
	Yes	-	0.41 (0.17)	-	-	0.60 (0.39)	-	-	0.58 (0.21)	-
	*P*	-	0.453	-	-	0.050	-	-	0.044	-
Xpert	No	0.44 (0.20)	0.40 (0.17)	<0.001	0.51 (0.41)	0.62 (0.39)	<0.001	0.44 (0.24)	0.55 (0.22)	<0.001
	Yes	-	0.41 (0.17)	-	-	0.62 (0.39)	-	-	0.58 (0.20)	-
	*P*	-	0.443	-	-	0.976	-	-	0.042	-
NAAT	No	0.44 (0.20)	0.40 (0.17)	<0.001	0.51 (0.41)	0.63 (0.39)	<0.001	0.44 (0.24)	0.56 (0.22)	<0.001
	Yes	-	0.41 (0.17)	-	-	0.61 (0.39)	-	-	0.57 (0.20)	-
	*P*	-	0.596	-	-	0.438	-	-	0.375	-
**Anti-TB treatment**										
Anti-TB treatment	No	0.44 (0.20)	0.42 (0.16)	0.230	0.51 (0.41)	0.61 (0.39)	0.014	0.44 (0.24)	0.61 (0.18)	<0.001
	Yes	-	0.40 (0.17)	-	-	0.63 (0.39)	-	-	0.56 (0.21)	-
	*P*	-	0.377	-	-	0.680	-	-	0.006	-
**Detailed anti-TB drugs’ usage**										
Isoniazid	No	0.44 (0.20)	0.41 (0.17)	<0.001	0.51 (0.41)	0.61 (0.39)	<0.001	0.44 (0.24)	0.61 (0.20)	<0.001
	Yes	-	0.40 (0.16)	-	-	0.63 (0.38)	-	-	0.54 (0.22)	-
	*P*	-	0.319	-	-	0.512	-	-	<0.001	-
Rifampicin	No	0.44 (0.20)	0.40 (0.17)	<0.001	0.51 (0.41)	0.63 (0.39)	<0.001	0.44 (0.24)	0.59 (0.21)	<0.001
	Yes	-	0.41 (0.16)	-	-	0.62 (0.38)	-	-	0.53 (0.21)	-
	*P*	-	0.160	-	-	0.596	-	-	<0.001	-
Pyrazinamide	No	0.44 (0.20)	0.40 (0.16)	<0.001	0.51 (0.41)	0.67 (0.38)	<0.001	0.44 (0.24)	0.57 (0.20)	<0.001
	Yes	-	0.41 (0.17)	-	-	0.60 (0.39)	-	-	0.56 (0.22)	-
	*P*	-	0.295	-	-	0.004	-	-	0.650	-
Ethambutol	No	0.44 (0.20)	0.42 (0.18)	<0.001	0.51 (0.41)	0.61 (0.39)	<0.001	0.44 (0.24)	0.61 (0.21)	<0.001
	Yes	-	0.40 (0.16)	-	-	0.63 (0.38)	-	-	0.54 (0.21)	-
	*P*	-	0.093	-	-	0.484	-	-	<0.001	-
Ofloxacin	No	0.44 (0.20)	0.40 (0.16)	<0.001	0.51 (0.41)	0.64 (0.38)	<0.001	0.44 (0.24)	0.55 (0.21)	<0.001
	Yes	-	0.43 (0.19)	-	-	0.57 (0.40)	-	-	0.61 (0.22)	-
	*P*	-	0.029	-	-	0.012	-	-	<0.001	-
Amikacin	No	0.44 (0.20)	0.40 (0.16)	<0.001	0.51 (0.41)	0.62 (0.38)	<0.001	0.44 (0.24)	0.55 (0.20)	<0.001
	Yes	-	0.42 (0.18)	-	-	0.62 (0.40)	-	-	0.60 (0.21)	-
	*P*	-	0.251	-	-	0.981	-	-	0.007	-
Vitamin B6	No	0.44 (0.20)	0.41 (0.16)	<0.001	0.51 (0.41)	0.63 (0.38)	<0.001	0.44 (0.24)	0.55 (0.21)	<0.001
	Yes	-	0.41 (0.19)	-	-	0.60 (0.41)	-	-	0.61 (0.21)	-
	*P*	-	0.551	-	-	0.434	-	-	0.002	-
Moxifloxacin	No	0.44 (0.20)	0.40 (0.16)	<0.001	0.51 (0.41)	0.62 (0.38)	<0.001	0.44 (0.24)	0.56 (0.20)	<0.001
	Yes	-	0.41 (0.19)	-	-	0.64 (0.40)	-	-	0.59 (0.22)	-
	*P*	-	0.585	-	-	0.401	-	-	0.048	-
Rifapentine	No	0.44 (0.20)	0.41 (0.16)	<0.001	0.51 (0.41)	0.62 (0.38)	<0.001	0.44 (0.24)	0.55 (0.20)	<0.001
	Yes	-	0.40 (0.19)	-	-	0.65 (0.39)	-	-	0.59 (0.22)	-
	*P*	-	0.357	-	-	0.170	-	-	0.027	-

*RPS20* Ribosomal Protein S20, *AP* alternative promoter, *KIF13B* Kinesin Family Member 13B, *SE* skipping exon, *UBE2B* Ubiquitin Conjugating Enzyme E2 B, *HC* healthy control, *TB* tuberculosis, *AIDS* acquired immune deficiency syndrome, *IGRA* interferon-γ release assay, *NAAT* nucleic acid amplification techniques

For continuous variables, correlation analyses were conducted, and no significant association was observed between the target AS event and the continuous variable (Supplementary Fig. S3a, b). Of note, we performed age-based subgroup analysis regarding the different age distribution between the TB and HC groups. We observed that the splicing differences of RPS20-exon1-AP, KIF13B-exon4-SE and UBE2B-exon7-SE between the TB and HC groups mainly came from the “20–40 years” (ΔPSI = −0.07, *P* < 0.001; ΔPSI = 0.14, *P* < 0.001; ΔPSI = 0.11, *P* < 0.001) and “40–60 years” subgroups (ΔPSI = −0.04, *P* = 0.003; ΔPSI = 0.11, *P* < 0.001; ΔPSI = 0.12, *P* < 0.001), but not the “<20 years” subgroup. In the “≥60 years” subgroup, both KIF13B-exon4-SE and UBE2B-exon7-SE were differentially spliced between TB patients and HCs (ΔPSI = 0.10, *P* = 0.030; ΔPSI = 0.16, *P* < 0.001) (Supplementary Fig. S3c). Mfuzz analysis indicated that in the HC group, KIF13B-exon4-SE was progressively inhibited and UBE2B-exon7-SE was gradually promoted with the increase of age. However, no obvious splicing trends were observed in the TB group, demonstrating that MTB invasion may disturb the life-cycle-dependent splicing pattern (Supplementary Fig. S3d).

### Predictive potential of AS biomarkers for TB progression and prognosis

In order to explore the utility of these AS biomarkers in predicting TB progression and prognosis, we searched for public TB-related sequencing datasets to validate these biomarkers (Supplementary Table S5).

**Fig. 3:**
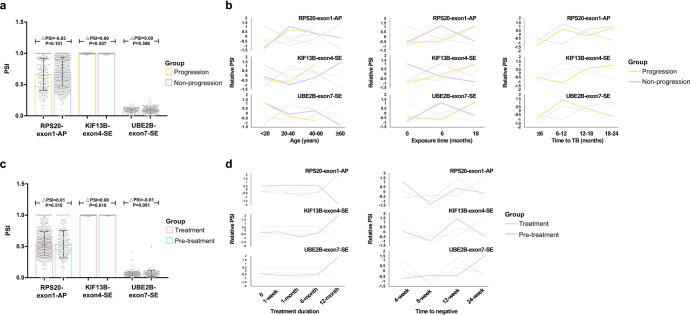
The splicing of target three events in the TB-progression and treatment cohorts. **a** Bar plots of the target three splicing events in the TB-progression cohort. **b** The Muffz analysis showing the splicing of three events over age, exposure time, and time to TB. **c** Bar plots of the three splicing events in the TB-treatment cohort. **d** The Muffz analysis showing the splicing of the target three events over treatment duration and time to negative. PSI percent spliced in, RPS20 Ribosomal Protein S20, AP alternate promoter, KIF13B Kinesin Family Member 13B, SE skipping exon, UBE2B Ubiquitin Conjugating Enzyme E2 B, TB tuberculosis

We combined four datasets that monitored patients for disease progression, with 214 samples in the progression group and 578 samples in the non-progression group. Regrettably, no meaningful splicing differences of all three biomarkers were found between the progression and non-progression groups (Fig. 3a), even after integrating age, sex, TB history, exposure time and TB-progression time into analysis (Fig. 3b and Supplementary Table S6).

We then analyzed other three datasets that contained samples taken from the patients before (*n* = 192) and after (*n* = 573) anti-TB treatment. The splicing of three biomarkers did not exhibit significant differences between the treatment and pre-treatment groups (Fig. 3c). No evident splicing pattern of these three biomarkers was found with the increase of treatment duration and time to negative (Fig. 3d). No statistical splicing difference was found among the TB patients with different treatment outcomes (Supplementary Table S7).

### Construction and validation of TB-related AS classifiers

**Fig. 4:**
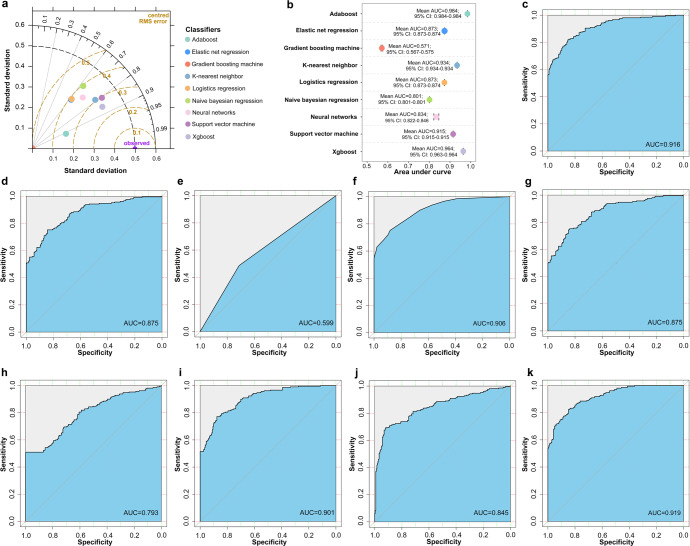
The performance of different classifiers. **a** Taylor diagram assessing the quality of classifier predictions against the reference in the training set. **b** Forest plot showing the results of bootstrapping. **c**–**k** Receiver operating characteristic curves of different classifiers (adaboost, elastic net regression, gradient boosting machine, K-nearest neighbor, logistics regression, naive bayesian regression, neural networks, support vector machine and xgboost, respectively) in the test set. Adaboost adaptive boosting, xgboost eXtreme gradient boosting, AUC area under curve, CI confidence interval

After clarifying the application scopes of these three biomarkers, we constructed TB-related classifiers based on three AS events and clinical variables. Altogether 2274 patients in the in-house cohort-2 were randomly divided into a training set (1820 participants) and a test set (454 participants). Among clinical variables, hematocrit was selected into a classifier regarding the highest odds ratio (Supplementary Tables S8, S9). Most classifiers presented promising area under curves of higher than 0.75 either in the internal validation or in the test set (Fig. 4). Of note, both adaptive boosting and neural networks achieved a specificity of 70% at a sensitivity of 90% in the test set, suggesting that these two classifiers surpassed the benchmark of World Health Organization-target product profile (Table 3). The assessment of modeling is listed in Supplementary Table S10.

**Table 3:** The performance of 9 classifiers in the test set

Classifiers	Sensitivity	Specificity	Specificity at 90% sensitivity	F1-score	AUC (95% CI)
Adaptive boosting	76%	89%	73%	0.808	0.916 (0.890–0.941)
Elastic net regression	72%	84%	60%	0.760	0.875 (0.843–0.907)
Gradient boosting machine	49%	71%	14%	0.534	0.599 (0.555–0.644)
K-nearest neighbor	75%	88%	66%	0.796	0.906 (0.879–0.932)
Logistics regression	74%	85%	61%	0.771	0.875 (0.843–0.906)
Naive bayesian regression	51%	100%	40%	0.675	0.793 (0.752–0.835)
Neural networks	76%	88%	70%	0.800	0.901 (0.874–0.928)
Support vector machine	75%	75%	45%	0.735	0.845 (0.808–0.882)
Xtreme gradient boosting	79%	86%	69%	0.809	0.919 (0.895–0.943)

*AUC* area under curve, *CI* confidence interval

### The regulation mechanism of target AS event

Considering the diagnostic ability of UBE2B-exon7-SE and the relationships between UBE2B-exon7-SE and clinical items, UBE2B-exon7-SE was selected as the target, and SRSF1 was predicted as a potential regulatory protein of this event (Supplementary Fig. S4).

**Fig. 5:**
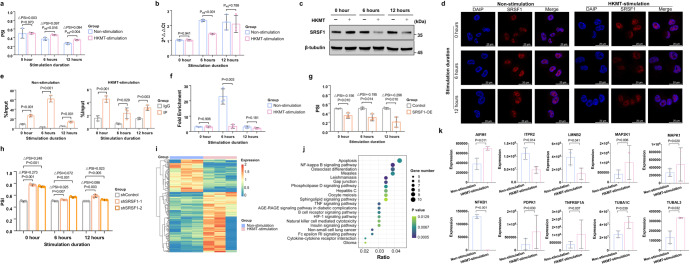
Regulation of SRSF1 on UBE2B-exon7-SE in tuberculosis. **a**, **b** qRT-PCR results showing the UBE2B-exon7-SE splicing and SRSF1 mRNA expression in macrophages with and without HKMT stimulation, respectively. (*n* = 3 biologically independent samples). Data are represented as mean ± standard deviation. *P*_adj_ was calculated by adjusting the splicing state of macrophages at the 0 h time point. **c** Western blotting results showing SRSF1 level in macrophages with and without HKMT stimulation. **d** Immunofluorescence staining of SRSF1 (red) and DAPI (blue) in macrophages. Scale bars for the images are 20 μm. **e** RNA immunoprecipitation showing the interaction between SRSF1 and UBE2B sequence. (*n* = 3 biologically independent samples). Data are represented as mean ± standard deviation. **f** RNA immunoprecipitation showing the alternations of UBE2B-SRSF1 binding caused by HKMT stimulation. (*n* = 3 biologically independent samples). **g**, **h** qRT-PCR results showing the splicing of UBE2B-exon7-SE in HKMT-stimulated control and SRSF1-OE, as well as shControl and shSRSF1. (*n* = 3 biologically independent samples). Data are represented as mean ± standard deviation. **i** Heatmaps showing the expression of differential ubiquitination proteins. **j** KEGG enrichment analysis of differential ubiquitination proteins. This plot showed the top 30 enriched pathways. **k** Bar plots showing the expression of differential ubiquitination proteins enriched in the apoptosis pathway. Data are represented as mean ± standard deviation. SRSF1 serine/arginine-rich splicing factor 1, UBE2B Ubiquitin Conjugating Enzyme E2 B, SE skipping exon, PSI percent spliced in, HKMT heat-killed *mycobacterium tuberculosis*, OE overexpression

Heat-killed MTB (HKMT)-stimulated macrophages had higher PSI values of UBE2B-exon7-SE than non-stimulated ones (ΔPSI_6h_ = 0.097, *P*_6h_ = 0.016; ΔPSI_12h_ = 0.084, *P*_12h_ = 0.004) (Fig. 5a). And SRSF1 expression significantly decreased after HKMT-stimulation (Fig. 5b, c). SRSF1 was located in the nucleus where AS occurred, and its subcellular locations did not change markedly after HKMT stimulation (Fig. 5d). The enrichment degree of UBE2B sequence in the IP group was starkly higher than that in the IgG group, indicating an interaction between SRSF1 and UBE2B sequence (Fig. 5e). Furthermore, the enrichment degree of UBE2B sequence reduced after HKMT stimulation, indicating a less interaction between SRSF1 and UBE2B sequence (*P*_6h_ = 0.003 and *P*_12h_ = 0.181) (Fig. 5f).

To verify the influence of SRSF1 on UBE2B-exon7-SE splicing, we constructed Tohoku Hospital Pediatrics-1 (THP-1) cell lines with SRSF1-overexpression (SRSF1-OE), and SRSF1-knockdown by using 2 short hairpin RNA (shRNA) (shSRSF1–1 and shSRSF1–2), respectively. Then, these cell lines were induced to be macrophages (Supplementary Fig. S5). The subcellular localization of SRSF1 did not change in SRSF1-OE and shSRSF1–1 macrophages after HKMT stimulation (Supplementary Fig. S6). Compared with control, HKMT-stimulated SRSF1-OE macrophages significantly promoted the skipping of UBE2B-exon7 (ΔPSI_0h_ = −0.156, *P*_0h_ = 0.010; ΔPSI_6h_ = −0.195, *P*_6h_ = 0.014; ΔPSI_12h_ = −0.296, *P*_12h_ = 0.010) (Fig. 5g). Compared with control, HKMT-stimulated shSRSF1–1 macrophages significantly inhibited the skipping of UBE2B-exon7 (ΔPSI_0h_ = 0.273, *P*_0h_ < 0.001; ΔPSI_6h_ = 0.025, *P*_6h_ = 0.007; ΔPSI_12h_ = 0.086, *P*_12h_ = 0.003). Similar results were also observed between HKMT-stimulated shControl and shSRSF1–2 macrophages (ΔPSI_0h_ = 0.248, *P*_0h_ < 0.001; ΔPSI_6h_ = 0.072, *P*_6h_ < 0.001; ΔPSI_12h_ = 0.023, *P*_12h_ = 0.005) (Fig. 5h).

### The biological roles of UBE2B-exon7-SE

UBE2B is a ubiquitin-conjugating enzyme, we thus detected ubiquitination proteome to preliminarily explore the effect of UBE2B-exon7-SE on protein ubiquitination and potential downstream targets. Altogether 231 ubiquitination proteins were differentially expressed between macrophages with and without HKMT stimulation (Fig. 5i). These differential proteins were closely associated with TNF signaling pathway, NF-kappa B signaling pathway, apoptosis and autophagy (Fig. 5j). Among the top 30 enriched pathways, the apoptosis pathway enriched the largest number of differentially expressed proteins (Fig. 5k).

## Discussion

In this paper, the splicing differences of three AS events between TB patients and HCs were identified based on large cohorts. Although such difference degrees appeared to be relatively limited, the biological effects of such changes should not be ignored. Due to the high expression of corresponding genes (UBE2B, S100A8, etc.), limited splicing changes can lead to obvious expression changes of relevant transcripts, thus generating significant biological effects. Subgroup analysis showed that the splicing of UBE2B-exon7-SE was independent of the influence of some confounding factors, emphasizing the potential performance stability of UBE2B-exon7-SE as a biomarker. Of note, the UBE2B-exon7-SE splicing differed between the TB patients with different treatment outcomes in the in-house cohort-1, but not in public datasets. Several reasons may explain this inconsistency. Included public datasets are generated based on the short read-length sequencing. To ensure the analysis algorithms’ consistency, we applied SUPPA2 to process these public datasets. The incompatibility between analysis algorithm and detection method may influence the result. Different prognostic criteria taken by ours and these public datasets also contributes to this inconsistency.^20^ Unfortunately, limited follow-up data in the in-house cohort-2 prevented us from further assessing the capacity of UBE2B-exon7-SE to predict TB-prognosis. Existing results suggest that UBE2B-exon7-SE may not be suitable for predicting TB prognosis. However, more validation is needed.

Pathogens can change the expression and subcellular location of SRSF to manipulate splicing.^19,21,22^ We found that HKMT stimulation changed SRSF1 expression rather than its localization. We observed that SRSF1 expression and the binding degree of SRSF1 and UBE2B sequence significantly declined after HKMT stimulation. This implies that in addition to expression, the binding capability of SRSF1 might contribute to its regulatory effect. However, it remains uncertain whether the change of binding capacity and the subsequent regulatory effects result directly from the HKMT stimulation, or are secondary to changes in expression.

We next wondered: what is the role of UBE2B-exon7-SE in TB? UBE2B is a member of the ubiquitin family, and it identifies the residue that is modified by ubiquitin and determines the type of substrate chain.^23^ The retention of exon7 triggers the early termination of protein translation, leading to the generation of H0YA80 protein with 138 amino acids. Alphaflod predicted the structure of H0YA80 protein, and revealed some potential variations of this isoform in the C-terminal compared to the P63146 protein (wild type) generated from the transcripts without exon7.^24–26^ The altered C-terminal may affect the binding of H0YA80 protein and ubiquitin, as well as the ability of ubiquitination modification for substrates.^27,28^ Our results also confirmed this to some extent. We found that the proteomic ubiquitination characteristics of macrophages changed significantly after HKMT stimulation, and most of the altered ubiquitination proteins were apoptosis-related proteins. Much is known about the engagement of ubiquitination-related molecules in TB,^29,30^ whilst this work proposed another potential approach whereby MTB regulated the splicing of UBE2B, then affecting the ubiquitination modification of apoptosis-related proteins and subsequent macrophage apoptosis. Further experiments are required to confirm the biological effects of this event.

This proof-of-concept study bridges biomarker development, large-scale cohort validation, and experimental studies to elucidate biological mechanism and clinical significance. However, this work still has some limitations. Both ONT sequencing and qPCR were used to detect AS events. However, qPCR might be unable to distinguish between transcripts that only differ by one base, even if primers are designed at junction sites. In other words, when targeting the same AS event, the relevant transcripts detected by qPCR might not one-to-one correspond to those detected by sequencing. Hence, a system that transforms the quantitative values of AS events given by different detection methods must be established. Furthermore, this work was carried out based on white blood cells and macrophages. We should also pay attention to the splicing of target AS events in different cell subtypes to comprehensively explore the MTB-host interactions at the splicing level.

In conclusion, alternative splicing is involved in the MTB-host interaction. The splicing of UBE2B-exon7-SE, RPS20-exon1-AP and KIF13B-exon4-SE could well indicate TB onset but not TB progression and prognosis. Notably, UBE2B-exon7-SE exhibited both excellent diagnostic capability and sufficient performance stability. Furthermore, we observed the arrest of SRSF1 and subsequently, inhibition of UBE2B exon7 skipping in macrophages stimulated with HKMT.

## Methods and materials

The research flow of this work is summarized in Fig. 1a.

### Cohort recruitment

The Clinical Trials and Biomedical Ethics Committee of West China Hospital, Sichuan University approved this study (Registration number of Chinese Clinical Trial Registry: ChiCTR1900028670). All participants provided informed consent.

An in-house cohort-1 and an in-house cohort-2 were consecutively recruited. For the in-house cohort-1, participants were enrolled from West China Hospital of Sichuan University from January 2020 to September 2020. TB patients in in-house cohort-1 need to meet the following criteria: (i) being diagnosed with TB according to the diagnostic guidelines;^31,32^ (ii) having no TB or anti-TB treatment history; (iii) did not receiving any anti-inflammatory treatment one month before recruitment; (iv) being free of other diseases; (v) aging more than 18 years; (vi) non-pregnant status. All enrolled TB participants would be followed up during their standard anti-TB treatment. The follow-up timepoints were set at the end of the intensive stage and whole treatment stage, respectively. Any patients who exhibited poor medication compliance, had the modified anti-TB treatment strategy or refused to continue to participate would be excluded. After six months follow-up, the treatment outcome was assessed based on the Guideline for primary care of pulmonary tuberculosis (2018).^33^ The reasons for the loss of follow-up were recorded. All HCs need to undergo TB-related detections (interferon gamma release assay and computed tomography) and only participants with negative results would be enrolled.

For the in-house cohort-2, participants were recruited from West China Hospital of Sichuan University, The Public Health Clinical Center of Chengdu, Ganzi People’s Hospital, and Zhaojue People’s Hospital of Liangshan Prefecture between January 2020 to December 2021. To balance the requirement of sample size and the progression of clinical recruitment, we took the above inclusion criteria (i) and (vi) as standards to enroll TB patients. The inclusion criteria of HCs were in line with those in the in-house cohort-1. PASS (v 16.0) was used to determine the minimum sample size.

### Data and sample collection

The following were recorded: demographic characteristics, personal history and complications, clinical symptoms, therapy strategy, image features, and laboratory detections. For items with missing value <30%, multiple imputation was performed by mice package (v 3.14.0).^34^ Items with missing value ≥30% were excluded from analysis.

Ethylene diamine tetraacetic acid-anticoagulated whole blood (5 ml) was collected from participants in both the in-house cohort-1 and the in-house cohort-2. After centrifugation and lysis of erythrocytes (QIAGEN), we extracted white blood cells. Then, we extracted total RNA using TRIzol reagent (Invitrogen).

### ONT sequencing and biomarker discovery

All steps were performed according to the ONT’s protocol. Briefly, strand-switch was used for reverse transcription. PCR amplification was realized by SQK-PCS109 PCR-cDNA kit (Oxford Nanopore Technologies), while barcodes were added by SQK-PBK004 rapid barcoding kit (Oxford Nanopore Technologies) to construct the sequencing library. PromethION (Oxford Nanopore Technologies) was applied to sequence. Base calling was conducted by Guppy (v 3.3.3) and raw reads with length<100 bp or quality<7 were filtered to obtain clean reads. Pychopper (v 1.0), Minimap 2 (v 2.16-r922) and Gffcompare (v 0.11.2) were applied to align clean reads and annotate consensus sequence, respectively. The human genome GRCh38 was used.

AS analysis and differential analysis were performed by SUPPA2 (v 2.3). Events with |ΔPSI| > 0.05 and *P* < 0.05 were identified as DASEs. For these DASEs, the expression of involved transcripts (transcripts per million>1), average PSI within a group (PSI > 0.01) and potential biological roles were further considered as the biomarker screening criteria.

### qRT-PCR and data procession

The reverse transcription was carried out by PrimeScript^MT^ RT reagent Kit with gDNA Eraser (TAKARA). qPCR was conducted by TB Green Fast qPCR Mix (TAKARA) and Appliedbiosystems QuantStudio 5. Two pairs of primers were designed to detect splice-in and splice-out transcripts, respectively (Supplementary Table S11). The relative expression of transcripts was calculated based on the 2^−ΔΔCt^ method and the PSI value was further yielded (PSI = 2^−ΔΔCt^_splice-in_/(2^−ΔΔCt^_splice-in_ + 2^−ΔΔCt^_splice-out_)). When detecting, investigators were blind to the grouping. Samples without corresponding Ct values were excluded.

### Subgroup analyses

Since complex phenotypes in the TB and HC groups can introduce bias of our results, subgroup analyses were performed to evaluate the influence of demographics and clinical characteristics. For categorical variables, we divided participants into different subgroups and compared the splicing differences of target AS events between different subgroups. For continuous variables, we set cutoffs according to the clinical and then divided participants into different subgroups to achieve a further comparison. Specifically, Mfuzz package (v 2.54.0) was used to exhibit the alternations of target events over some time-dependent continuous variables (age, treatment duration, etc.).

### Public datasets retrieval and analysis

To evaluate the value of developed biomarkers in predicting TB progression and prognosis, and further clarify the optimum application scope of these biomarkers, we constructed TB-progression and treatment cohorts based on public datasets, respectively. TB-progression and treatment related sequencing datasets were searched in January 2021 from European Bioinformatics Institute ArrayExpress, NCBI Gene Expression Omnibus database, and NCBI Sequence Read Archive. The retrieval keywords were “tuberculosis” or “TB”. Datasets that generated by detecting whole blood samples of homo species and provided raw data were included.

The information of included datasets (sample size, detection platform, participant characteristics, etc.) were recorded. Raw files of each included sample were downloaded and filtered by trim_galore (v 0.6.7) to obtain clean reads (length>36 bp and quality>25). The alignment and AS analysis were achieved by salmon (v 0.14.1) and SUPPA2 (v 2.3), respectively. The human genome GRCh38 was used. For events detected in ONT sequencing but not the included high-throughput sequencing datasets, the PSI values of corresponding events were manually calculated based on the expression of involved transcripts. Samples without the PSI values of target events were excluded.

### Variable selection and classifier construction

Classifiers were designed according to Transparent Reporting of a multivariable prediction model for Individual Prognosis Or Diagnosis.

The participants within the in-house cohort-2 were randomly divided into a training set and a test set by Base package in R (v 4.1.2) according to the ratio of 8:2. Inputted variables included AS biomarkers and clinical items selected by logistics regression (stats package, v 4.1.2). Candidate clinical items included basic demographic characteristics (age, sex, etc.), and indicators that were detected based on Ethylene diamine tetraacetic acid-anticoagulated whole blood (the same sample as the splicing detection). For all inputted variables, variance inflation factor was used to assess the multicollinearity. If variance inflation factor was less than 10, there was no multicollinearity among variables.

Nine machine learning classifiers (adaptive boosting, elastic net regression, gradient boosting machine, K-nearest neighbor, logistics regression, naive bayesian regression, neural networks, support vector machine, and eXtreme gradient boosting) were built by Adabag (v 4.2), glmnet (v 4.1–3), gbm (v 1.6.8), caret (v 6.0–90), glmnet (v 4.1–3), e1071 (v 1.7–9), neuralnet (v 1.44.2), e1071 (v 1.7–9), and xgboost packages (v 1.6.0.1) in R, respectively.

In the training set, bootstrap (boot package, v 1.3–28) was used for internal validation and the number of bootstrap replicates was set to 1000 times. Youden index was used as the threshold value. Sensitivity, specificity, and area under curve were yielded to quantitatively evaluate the classifier performance. Specificity at 90% sensitivity was also calculated to assess whether the classifiers’ performance meets the requirements of World Health Organization-target product profile for new TB diagnostic test.^35^ Taylor diagrams, forest plots and receiver operating characteristic curve were plotted for visualization.

### Cell culture, induction and stimulation

The human myeloid leukemia mononuclear cells, THP-1-derived macrophages are commonly used to construct TB-associated models in vitro. Therefore, they were selected for this work.

THP-1 cell line was a gift from Dr. Xuan Chen (West China Hospital of Stomatology, Sichuan University) and further identified by short tandem repeat loci identification (Supplementary Fig. S7). THP-1 cells and THP-1-derived macrophages were cultured in RPMI 1640 medium (Gibco) supplemented with 10% fetal bovine serum (Gibco). HEK293T cells were cultured in DMEM medium (Gibco) supplemented with 10% FBS (Gibco). Cells were cultured at 37 °C with 5% CO_2_.

To obtain macrophages, THP-1 cells were differentiated by using 100 ng/ml phorbol ester (APExBIO) for 24 h, followed by a recovery period of 24 h in fresh medium without PMA. Then, HKMT (InvivoGen) was used to stimulate THP-1-derived macrophages according to previous protocols.^36,37^ The concentration and duration of HKMT stimulation were set to 10 µg/ml, and 0, 6, and 12 h, respectively.

### Target AS biomarker selection and potential SRSF protein prediction

The AS biomarker that exhibited excellent diagnostic capacity and performance stability (UBE2B-exon7-SE) was selected as a target. RBPsuite, CatRAPID and SpliceAid2 were applied to predict potential SRSF proteins. The predicted results of these three databases were intersected and candidate SRSF proteins were further filtered by the binding sites (exon7). In this work, SRSF1 protein was predicted as the candidate.

### Western blotting

Cells were washed with phosphate buffer saline (PBS) (Solarbio) and resuspended with RIPA cell lysis buffer (Solarbio) containing 1% phenylmethylsulfonyl fluoride (Solarbio) and 1% protease inhibitor cocktail (Sigma-Aldrich), incubated for 30 min on ice followed by centrifuged at 4 °C, 12,000 *g* for 15 min to collect supernatants. BCA Protein Assay Kit (Solarbio) was used to determine protein concentration. Supernatants were mixed with 5X loading buffer (Solarbio) and incubated at 100 °C for 5 min. Samples were resolved by SDS-PAGE and transferred to a PVDF membrane. The following antibody were used: β-tubulin (Beijing Zhong Shan Goldenbridge Biotechnology #TA-10, 1:1000), SRSF1 (Santa Cruz Biotechnology #sc-33652, 1:100) and horseradish peroxidase-labeled anti-mouse IgG antibody (Beijing Zhong Shan Goldenbridge Biotechnology #ZB-2305, 1:5000).

### Immunofluorescence staining

Cells were fixed with 4% paraformaldehyde (Leagene Biotechnology) for 15 min and washed with PBS (Solarbio) for 3 times (1 min for each time). Then, cells were permeabilized with 0.5% Triton X-100 (Dimond) for 20 min at room temperature and washed with PBS. Next, cells were blocked with 5% bovine serum albumin V (Solarbio) for 30 min at room temperature and incubated with SRSF1 antibody (Santa Cruz Biotechnology #sc-33652, 1:50) in a wet box at 4 °C overnight. Then, cells were washed with PBS, followed by be incubated with fluorescent IgG antibody (Alexa Fluor 594 conjugated, Invitrogen #A21203, 1:1000, red) in a dark room for 1 h. Again, after washing with PBS, cells were incubated with 4’,6-diamidino-2-phenylindole (Solarbio #C0060, 10 µg/ml) in a dark room for 5 min, followed by washing with PBS. Finally, an antifade mounting medium (Beyotime Biotechnology) was added, and a confocal laser scanning microscope (Leica) was used to capture the fluorescence signal.

### RNA immunoprecipitation

To examine the interaction of SRSF1 protein and the target sequence, RNA immunoprecipitation was conducted and all steps were performed according to the manufacturer’s protocol (BerSinBio# Bes5101). In short, cells were wash with PBS and resuspended with lysis buffer containing protease inhibitors and RNA enzyme inhibitors (lysis buffer: protease inhibitors: RNA enzyme inhibitors = 1700:17:7.5); then, the DNA removal process was carried out. Cell lysate was divided into three groups (input, IP, and IgG groups). Anti-SRSF1 antibodies (Santa Cruz Biotechnology #sc-33652, 5 μg) and IgG antibody (BerSinBio#Bes5101, 5 μg) were incubated with cell lysates of IP and IgG groups, respectively, at 4 °C for 16 h. Next, the RNA-protein complexes were isolated by incubating cell lysates with the protein A/G magnetic beads at 4 °C for 1 h. After proteinase K digestion, protein-bound RNAs were extracted by phenol/chloroform/isoamyl alcohol (125:24:1) (Solarbio). The protein-bound RNAs were detected by qRT-PCR and assessed by %Input (%Input = 2^−[ΔCtIP-(ΔCtinput-log2 Input Dilution Factor)]^, Input Dilution Factor = (Volume _Input_/Volume _Input_ + Volume _IP_ + Volume _IgG_)^−1^). The fold enrichment (fold enrichment = 2^−(ΔCtIP-ΔCtIgG)^) was also calculated to evaluate the alternations of RNA-protein binding caused by HKMT stimulation. The primers used in this assay are listed in Supplementary Table S11.

### Stable knockdown and overexpressed cells construction

For overexpression, a human SRSF1 cDNA clone was fused to pCDH-CMV- MCS-EF1-Puro vector in the EcoRI and NotI sites. For knockdown, the pLKO.1-puro based lentiviral shRNA was constructed. The following shRNA oligo sequences were used: (i) shSRSF1–1: CCGGGCATCTACGTGGGTAACTTACCTCGAGGTAAGTTACCCACGTAGATGCTTTTTG; (ii) shSRSF1–2: CCGGGCAGAGGATCACCACGCTATTCTCGAGAATAGCGTGGTGATCCTCTGCTTTTTG. Lentivirus was prepared in HEK293T cells by transfecting lentiviral plasmid with packaging plasmids. After 6–8 h of transfection, a fresh medium was changed. After 48 h of transfection, THP-1 cells were infected with 0.45 μm filtered viral supernatant and 8 μg/ml polybrene was also used. After 24 h of infection, a fresh medium was changed. After 48 h of infection, puromycin selection (1 μg/ml) was performed for 6 days, and then collected for subsequent analyses. Overexpression and knockdown were validated by western blotting.

### Mass-spectrometric quantification of ubiquitination proteome

THP-1-derived macrophages with and without HKMT stimulation were collected and lysed to extract protein. After quality assessment, Trypsin Gold (Promega) and anti-Ubiquitin Remnant Motif (K-ε-GG) beads (Cell Signaling Technology) were used for the peptide preparation and K-ε-GG enrichment of peptides, respectively. EASY-nLCTM 1200 UHPLC system (Thermo Fisher) and an Orbitrap Q Exactive HF-X mass spectrometer (Thermo Fisher) were applied for detection.

Based on UniProt database, Proteome Discoverer (V 2.4) was used to search the resulting spectra from each fraction. Protein containing at least one unique peptide was identified at false discovery rate less than 1.0% on peptide and protein level, respectively. Protein quantification and differential analysis (fold change≥1.5 or ≤0.67; *P* < 0.05) were carried out by the intensity-based precursor quantification and T-test, respectively.

### Statistical analysis

Data analyses were conducted using R (v 4.1.2) and GraphPad Prism (v 7.0a). Data was expressed as the proportion, mean ± standard deviation or median (percent_25_ and percent_75_). Heatmaps, circos plots, violin plots, pirate plots and histograms were drawn to visualize data. The difference between groups was tested by Student’s *t* test, ANOVA test or Kruskal-Wallis test. A *P* < 0.05 (two-side) was considered significant.

## Supplementary information

Supplementary Material

STARD-2015-Checklist

## Data Availability

Raw files of ONT sequencing were uploaded into China National Center for Bioinformation/Beijing Institute of Genomics, Chinese Academy of Sciences (https://ngdc.cncb.ac.cn: accession no. HRA003134). Raw files of ubiquitination proteome were uploaded into ProteomeXchange (http://proteomecentral.proteomexchange.org/cgi/GetDataset: accession no. PXD039906). High-throughput sequencing datasets used in this work are public and their dataset IDs are provided in Supplementary Table S5. Raw data of in vitro experiments are available from the corresponding author upon request.

## Code availability

All codes used in this work are uploaded on Github (https://github.com/MengyuanLyu/TB-diagnostic-AS-biomarker).
