# Supplementary material for: From tuberculosis bedside to bench: UBE2B splicing as a potential biomarker and its regulatory mechanism
Source: Signal Transduct Target Ther. 2023 Feb 24;8:82. doi: 10.1038/s41392-023-01346-2 (PMC9958017; doi:10.1038/s41392-023-01346-2)
Supplement: Supplementary file 1 — Supplementary Material [file 41392_2023_1346_MOESM1_ESM.docx]

Supplementary Materials for

From tuberculosis bedside to bench: UBE2B splicing as a potential biomarker and its regulatory mechanism

Mengyuan Lyu, Jian Zhou, Yanbing Zhou, Weelic Chong, Wei Xu, Hongli Lai, Lu Niu, Yang Hai, Xiaojun Yao, Sheng Gong, Qinglan Wang, Yi Chen, Yili Wang, Liyu Chen, Zewang Gema, Jiongjiong Zeng, Chengdi Wang, Binwu Ying^✉^

Correspondence to: [yingbinwu@scu.edu.cn](mailto:yingbinwu@scu.edu.cn)

**This PDF file includes:**

Supplementary Figure S1 to S7

Supplementary Table S1 to S11

**
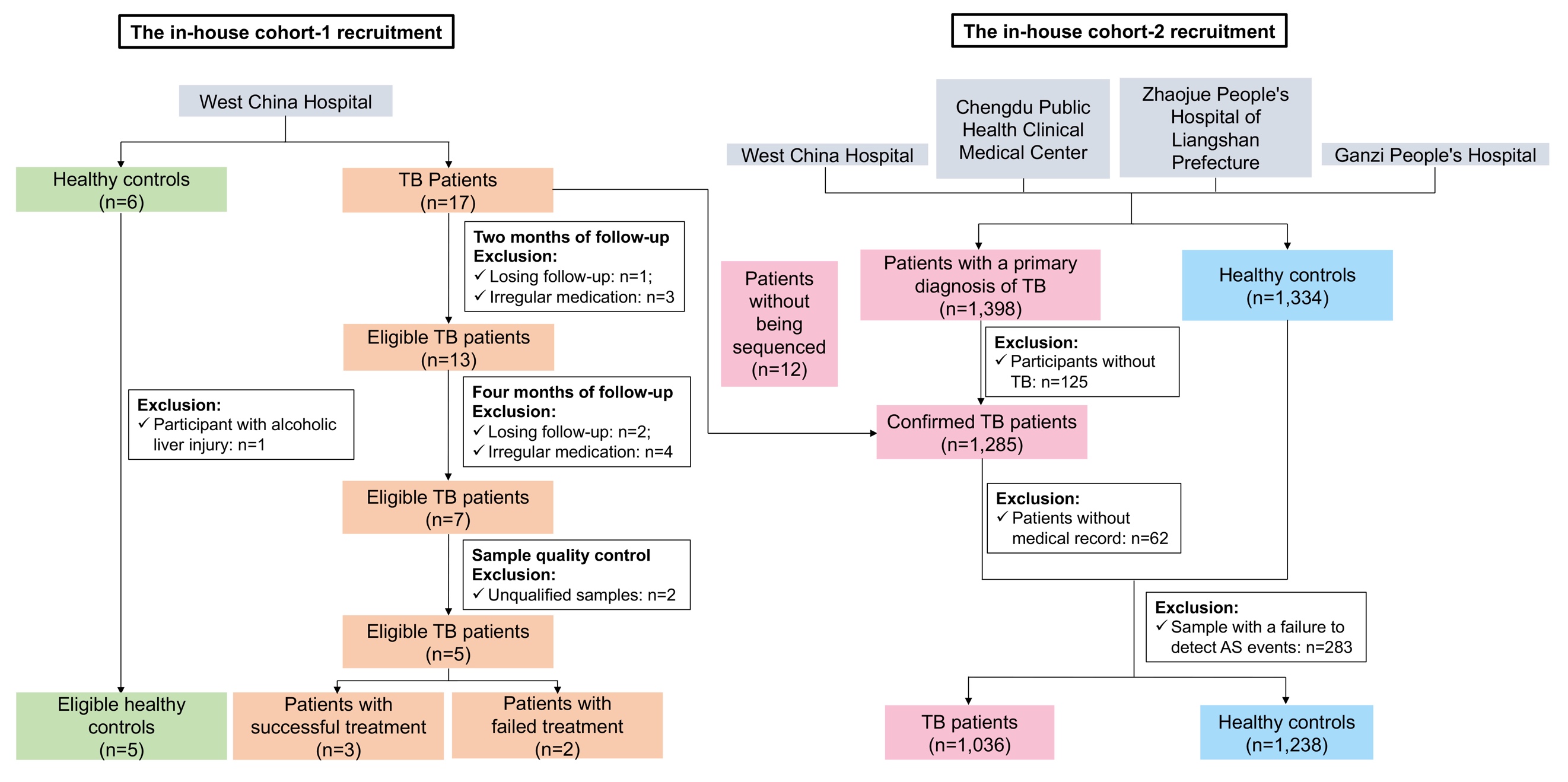
Supplementary Figure S1.** Flowchart of the participant recruitment. TB tuberculosis, AS alternative splicing.


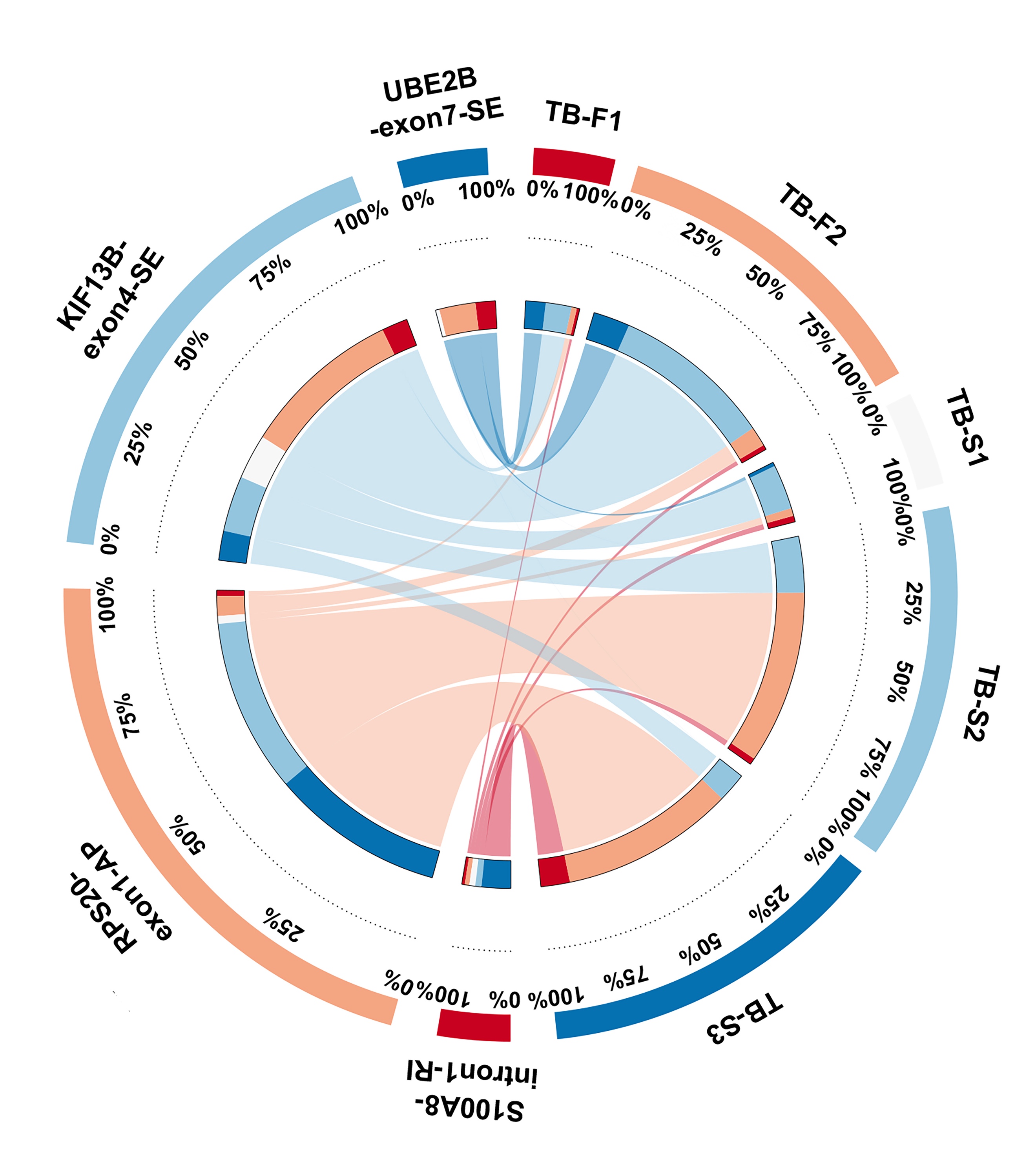


**Supplementary Figure S2.** Circus plots showing the splicing of selected four splicing events in tuberculosis patients with different prognosis. TB-F anti-tuberculosis treatment failure, TB-S anti-tuberculosis treatment success, S100A8 S100 Calcium Binding Protein A8, RI intron retention, RPS20 Ribosomal Protein S20, AP alternate promoter, KIF13B Kinesin Family Member 13B, SE skipping exon, UBE2B Ubiquitin Conjugating Enzyme E2 B.

**
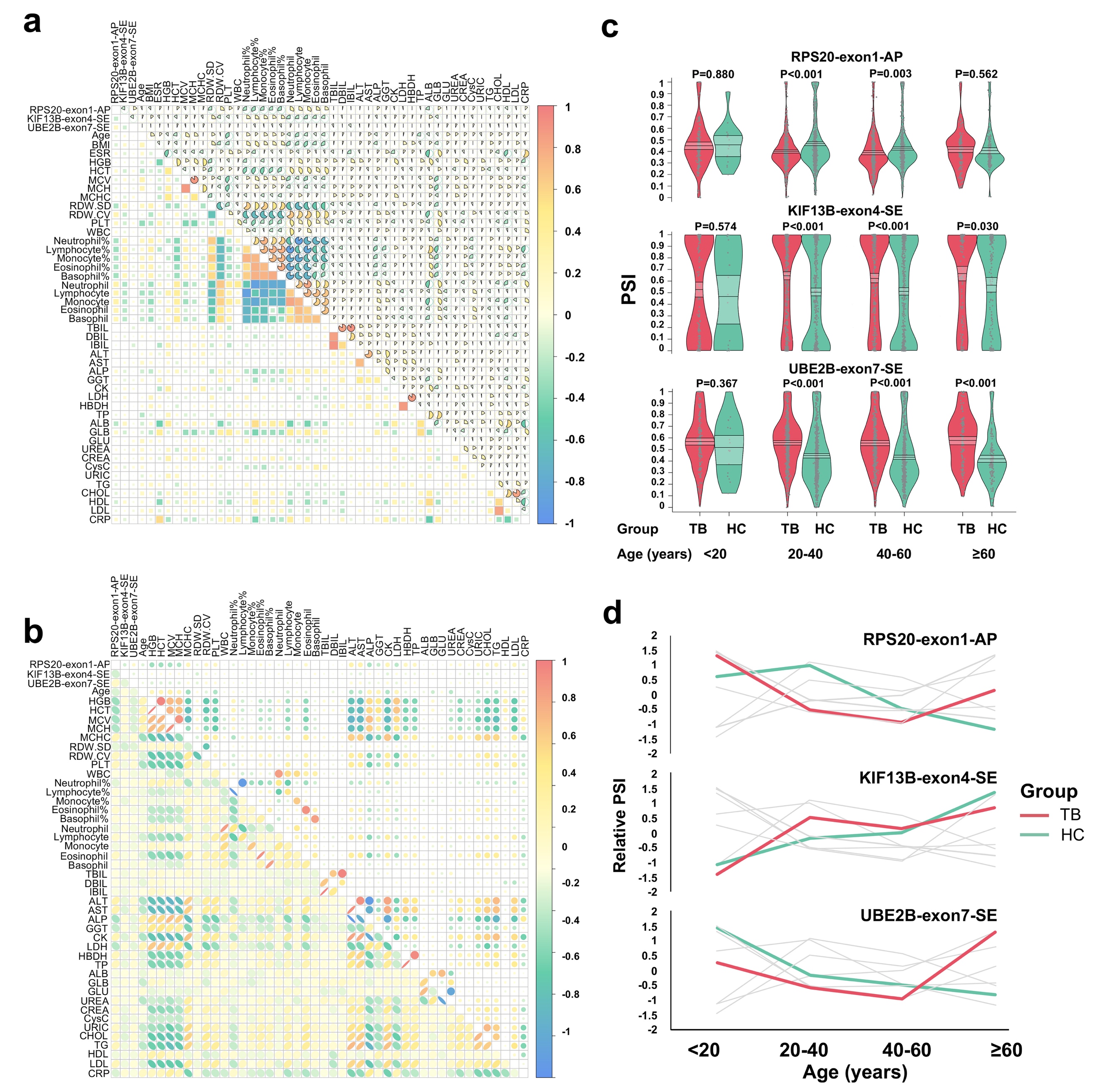
**

**Supplementary Figure S3.** Relationships between target alternative splicing events and continuous variables.

**a and b** Correlation between target alternative splicing events and continuous variables in the tuberculosis and healthy control groups, respectively. Sector, square, circle and ellipse size indicated the correlation strength. **c** The splicing of target events in different age subgroups. There were 151, 438, 312 and 135 tuberculosis patients in the “<20 years”, “20-40 years”, “40-60 years” and “≥60 years”subgroups, respectively. While there were 17, 531, 542 and 148 healthy controls in the “<20 years”, “20-40 years”, “40-60 years” and “≥60 years”subgroups, respectively. **d** Muffz analysis showing the splicing of 3 events over age. RPS20 Ribosomal Protein S20, AP alternative promoter, KIF13B Kinesin Family Member 13B, SE skipping exon, UBE2B Ubiquitin Conjugating Enzyme E2 B, BMI body mass index, ESR erythrocyte sedimentation rate, HGB hemoglobin, HCT hematocrit, MCV mean corpuscular volume, MCH mean corpuscular hemoglobin, MCHC mean corpuscular hemoglobin concentration, RDW.SD red cell distribution width, RDW.CV red blood cell distribution width-variation coefficient, PLT platelet, WBC white blood cell, TBIL total bilirubin, DBIL direct bilirubin, IBIL indirect bilirubin, ALT alanine aminotransferase, AST glutamic oxaloacetic transaminase, ALP alkaline phosphatase, GGT gamma-glutamyl transpeptidase, CK creatine kinase, LDH lactate dehydrogenase, HBDH hydroxybutyrate dehydrogenase, TP total protein, ALB albumin, GLB globulin, CREA creatinine, CysC cystatin C, URIC uric acid, TG triglyceride, CHOL cholesterol, HDL high-density lipoprotein cholesterol, LDL low-density lipoprotein cholesterol, CRP C-reactive protein, PSI percent spliced in, TB tuberculosis, HC healthy control.


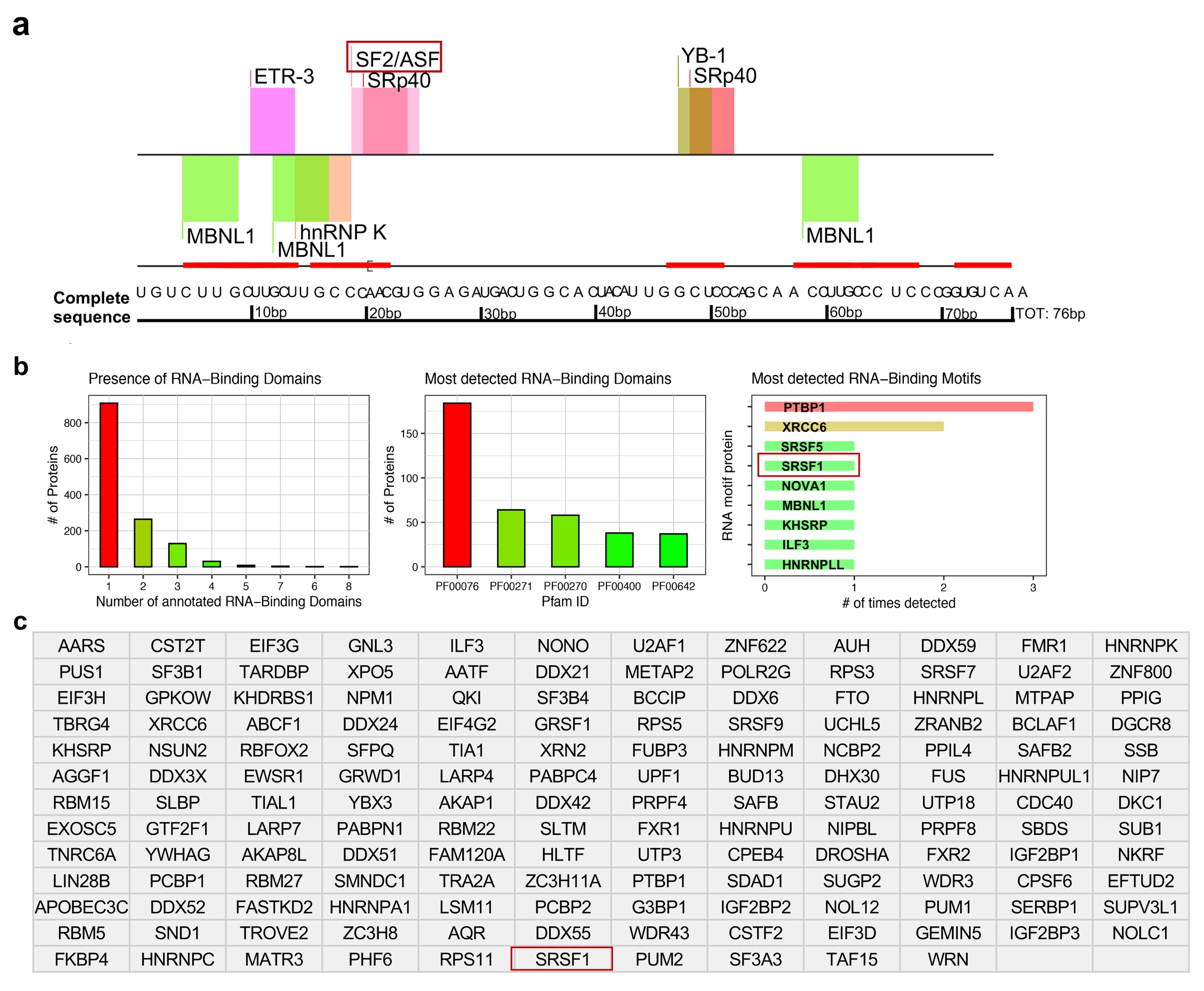


**Supplementary Figure S4.** Prediction of the potential SRSF protein.

**a-c** Candidate SRSF proteins predicted by SpliceAid2, CatRAPID, and RBPsuite, respectively. Candidates that were bound to the sequence of UBE2B exon7 were shown. SRSF serine/arginine-rich splicing factor, UBE2B Ubiquitin Conjugating Enzyme E2 B.


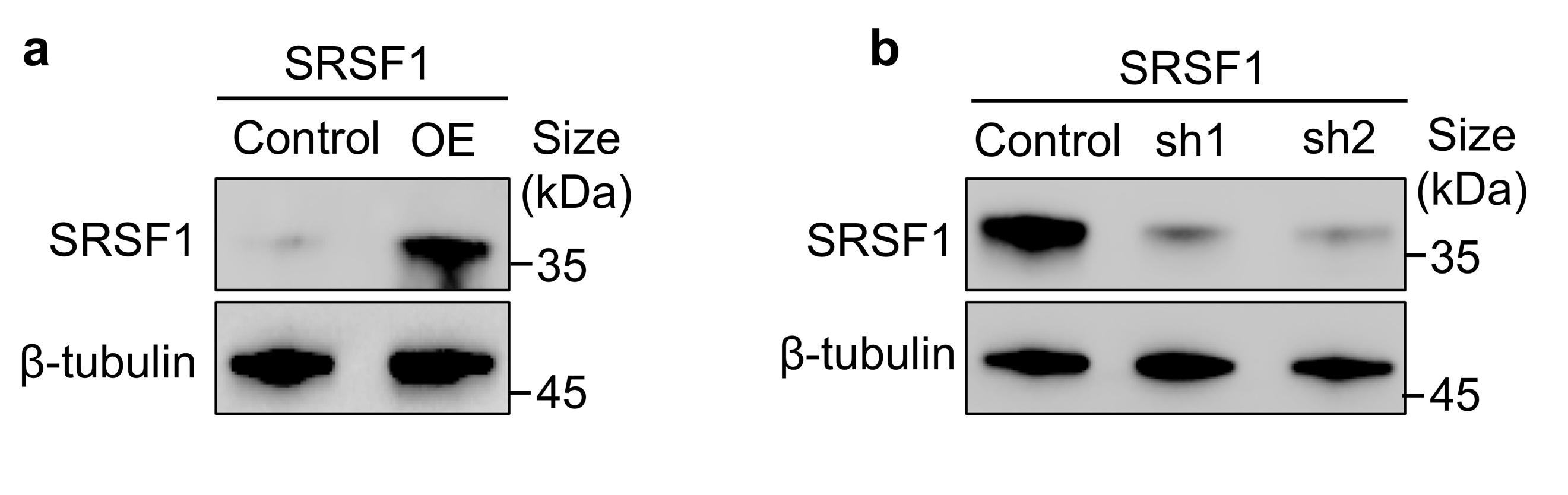


**Supplementary Figure S5.** SRSF1 expression in macrophages.

**a** Western blotting showing SRSF1 expression in SRSF1-OE and control. **b** Western blotting showing SRSF1 expression in shSRSF1 and control. Three independent experiments were repeated. SRSF1 serine/arginine-rich splicing factor 1, HKMT heat-killed *mycobacterium tuberculosis*, OE overexpression.


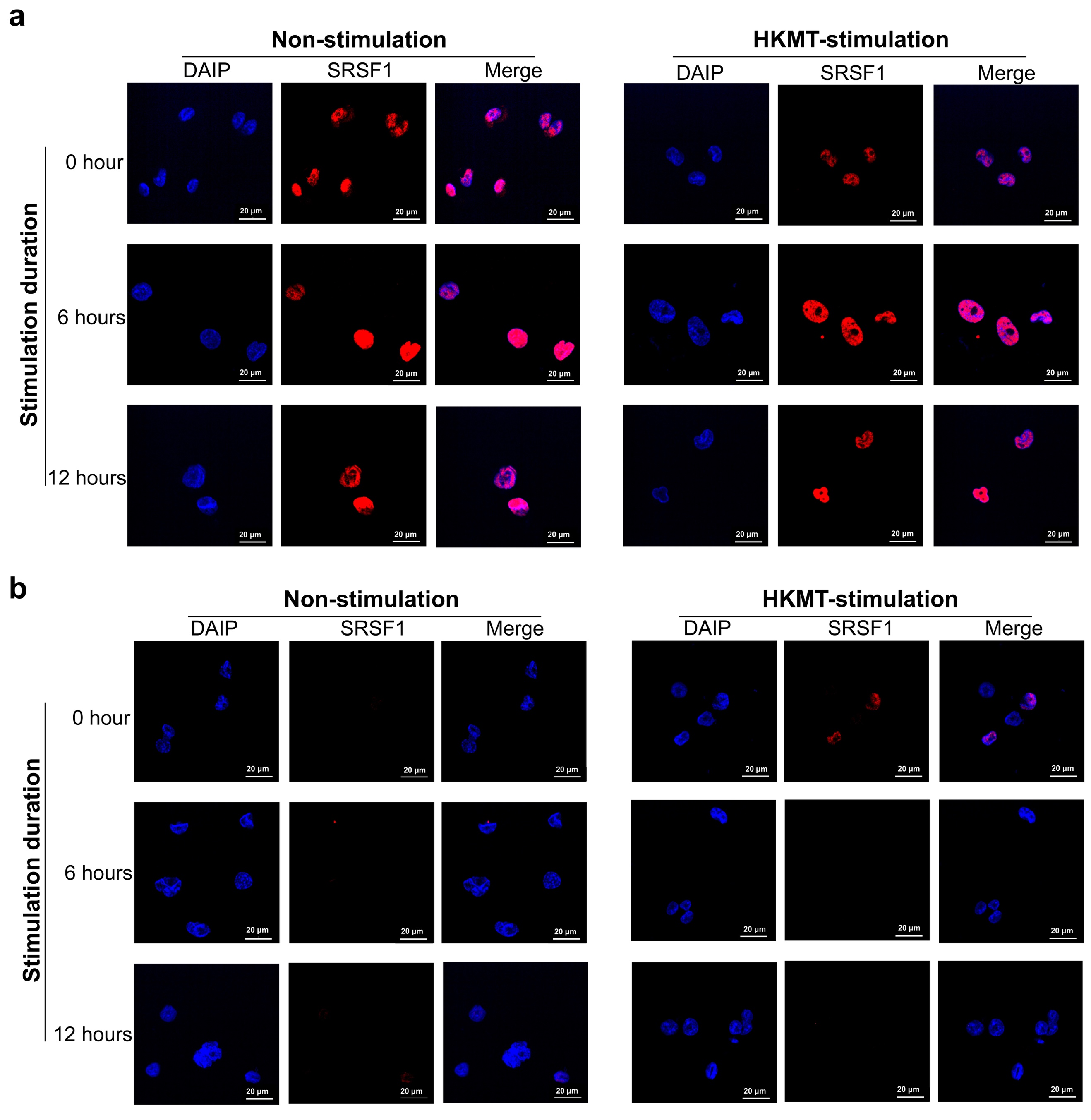


**Supplementary Figure S6.** SRSF1 subcellular location in different macrophages.

**a** Immunofluorescence staining of SRSF1 (red) and DAPI (blue) in SRSF1-OE macrophages with or without HKMT stimulation. **b** Immunofluorescence staining of SRSF1 (red) and DAPI (blue) in shSRSF1-1 macrophages with or without HKMT stimulation. Scale bars for the images are 20 μm. SRSF1 serine/arginine-rich splicing factor 1, HKMT heat-killed *mycobacterium tuberculosis*.


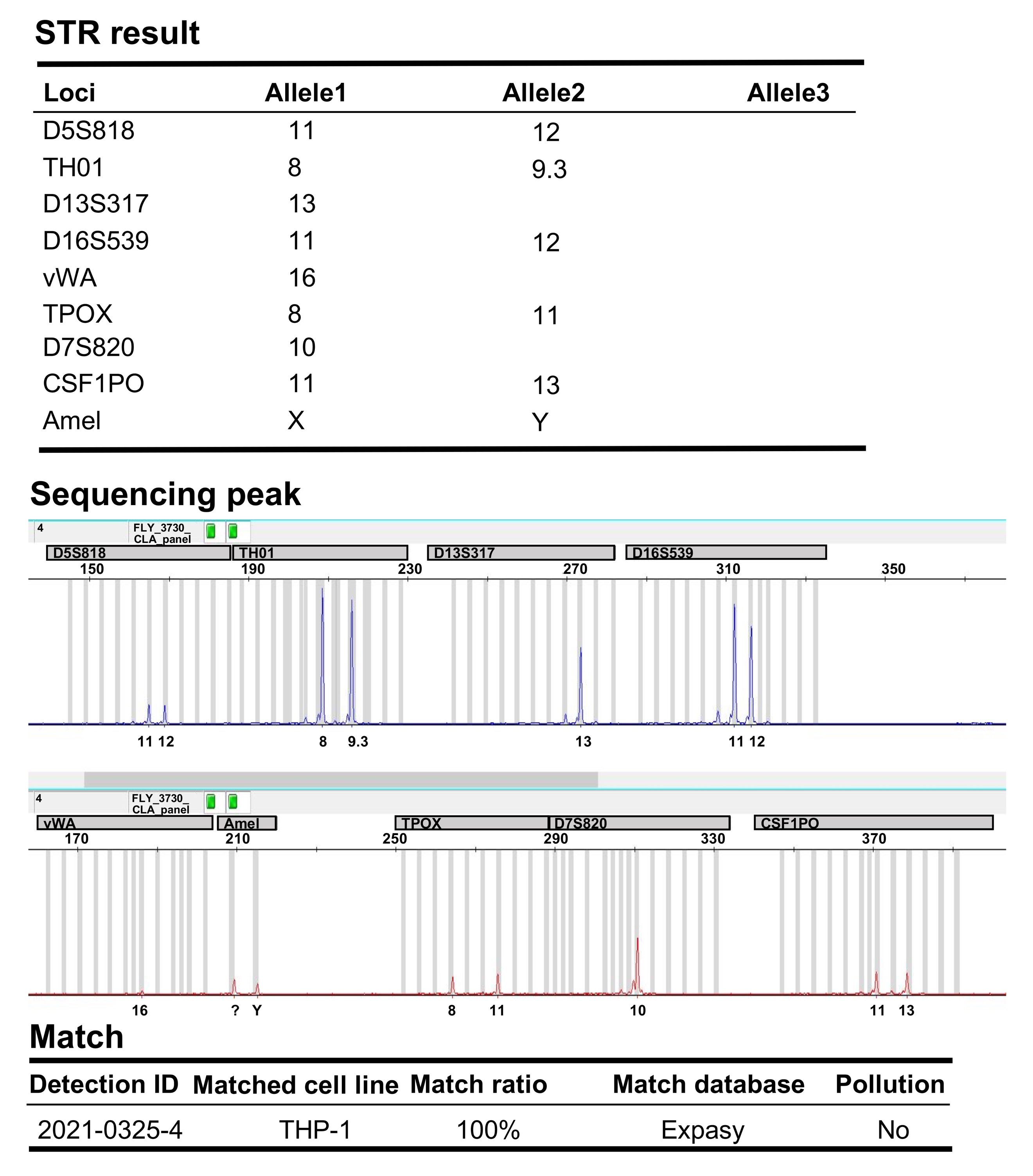


**Supplementary Figure S7.** STR identification of THP-1 cell. STR short tandem repeat, THP-1 Tohoku Hospital Pediatrics-1.

**Supplementary Table S1.** The characteristics of the in-house cohort-1.

| **Characteristics** | **Healthy controls (n=5)** | **TB patients**  **(n=5)** | **P** |
| --- | --- | --- | --- |
| **Demographic characteristics, *mean±standard deviation or number*** | | | |
| Age, years | 34.00±9.00 | 32.00±3.00 | 0.598 |
| Sex (Female/Male) | 3/2 | 2/3 | 1.000 |
| TB history (Yes/No) | 0/5 | 0/5 | - |
| TB contact history (Yes/No) | 0/5 | 2/3 | 0.444 |
| **Clinical symptoms, *n (%)*** | | | |
| Cough | 0 (0.00) | 4 (80.00) | 0.048 |
| Expectoration | 0 (0.00) | 2 (40.00) | 0.444 |
| Weight loss | 0 (0.00) | 2 (40.00) | 0.444 |
| Chest tightness | 0 (0.00) | 2 (40.00) | 0.444 |
| Insomnia | 0 (0.00) | 1 (20.00) | 1.000 |
| Hemoptysis | 0 (0.00) | 1 (20.00) | 1.000 |
| **Imaging characteristics, *n (%)*** | | | |
| Pulmonary GGO | 0 (0.00) | 1/4 | - |
| Pulmonary cavity | 0 (0.00) | 1/4 | - |
| Pulmonary nodule | 0 (0.00) | 2/3 | - |
| Pulmonary patchy shadow | 0 (0.00) | 3/2 | - |
| Pulmonary calcification | 0 (0.00) | 1/4 | - |
| **Tuberculosis-related laboratory detections, *n (%)*** | | | |
| TB-DNA | 0 (0.00) | 1 (20.00) | - |
| Smear | 0 (0.00) | 4 (80.00) | - |
| Culture | 0 (0.00) | 2 (40.00) | - |
| **Laboratory examinations,** ***mean±standard deviation or median (percent_25_- percent_75_).*** | | | |
| ***Whole blood-based detections*** | | | |
| ESR, mm/h | 9.67±6.51 | 13.33±10.26 | 0.629 |
| Hemoglobin, g/L | 144.80±20.58 | 142.75±21.24 | 0.888 |
| Hematocrit, L/L | 0.43±0.04 | 0.43±0.05 | 0.871 |

**Continued**

| **Characteristics** | **Healthy controls (n=5)** | **TB patients**  **(n=5)** | **P** |
| --- | --- | --- | --- |
| MCV, fL | 92.10 (81.85-94.00) | 92.10 (88.23-98.68) | 1.000 |
| MCH, pg | 31.50 (26.30-32.45) | 30.50 (29.75-32.68) | 0.905 |
| MCHC, g/L | 334.80±16.45 | 333.00±14.11 | 0.867 |
| RDW.SD, fL | 40.66±2.58 | 42.60±3.25 | 0.349 |
| RDW.CV, % | 12.62±1.16 | 12.60±0.49 | 0.976 |
| Platelets, 10^8^/L | 237±35.30 | 190±35.62 | 0.085 |
| White blood cell, 10^9^/L | 5.75±1.35 | 5.92±0.66 | 0.828 |
| Neutrophil, 10^9^/L | 3.01±1.03 | 3.34±0.57 | 0.592 |
| Lymphocyte, 10^9^/L | 2.23±0.47 | 2.14±0.23 | 0.712 |
| Monocyte, 10^9^/L | 0.34 (0.28-0.51) | 0.32 (0.27-0.37) | 0.730 |
| Eosinophil, 10^9^/L | 0.08±0.05 | 0.10±0.04 | 0.671 |
| Basophil, 10^9^/L | 0.05±0.03 | 0.03±0.02 | 0.385 |
| Neutrophil, % | 51.44±7.03 | 56.15±4.22 | 0.280 |
| Lymphocyte, % | 39.72±7.83 | 36.23±3.70 | 0.442 |
| Monocyte, % | 6.60±1.59 | 5.40±0.93 | 0.225 |
| Basophil, % | 0.84±0.37 | 0.60±0.42 | 0.395 |
| ***Serum-based detections*** | | | |
| TBIL, µmol/L | 15.96±3.48 | 13.94±6.68 | 0.565 |
| DBIL, µmol/L | 3.94±0.99 | 4.70±2.41 | 0.533 |
| IBIL, µmol/L | 12.02±2.56 | 9.24±4.49 | 0.263 |
| ALT, IU/L | 14.00 (11.00-36.00) | 18.00 (11.50-33.85) | 1.000 |
| AST, IU/L | 20.20±6.46 | 23.36±7.30 | 0.489 |
| AST/ALT | 1.16±0.44 | 1.28±0.50 | 0.694 |
| ALP, IU/L | 72.20±14.33 | 70.40±9.55 | 0.821 |
| GGT, IU/L | 12.00 (10.50-19.50) | 17.00 (8.50-45.00) | 0.421 |
| Creatine kinase, IU/L | 98.40±16.80 | 87.75±44.06 | 0.630 |
| LDH, IU/L | 170.00±22.71 | 155.50±16.22 | 0.320 |

**Continued**

| **Characteristics** | **Healthy controls (n=5)** | **TB patients**  **(n=5)** | **P** |
| --- | --- | --- | --- |
| HBDH, IU/L | 131.40±20.90 | 123.40±10.64 | 0.468 |
| Total Protein, g/L | 74.46±3.90 | 75.70±7.11 | 0.741 |
| Albumin, g/L | 48.44±3.05 | 46.06±7.98 | 0.560 |
| Globulin, g/L | 26.02±2.79 | 29.64±3.18 | 0.092 |
| Albumin/Globulin | 1.88±0.25 | 1.58±0.37 | 0.165 |
| Glucose, mmol/L | 4.88±0.90 | 4.38±0.50 | 0.299 |
| Urea, mmol/L | 3.98±0.35 | 4.24±0.63 | 0.449 |
| Creatinine, mmol/L | 72.00±13.04 | 74.80±17.05 | 0.778 |
| Cystatin C, mg/L | 0.73 (0.69-0.81) | 0.71 (0.41-0.90) | 0.730 |
| Triglycerides, mmol/L | 1.06±0.30 | 0.59±0.23 | 0.023 |
| HDL, mmol/L | 1.45±0.21 | 1.56±0.33 | 0.580 |
| LDL, mmol/L | 2.82±0.58 | 1.97±0.58 | 0.050 |
| CRP, mmol/L | 2.40± - | 0.91±0.78 | 0.362 |

*TB* tuberculosis, *GGO* ground-glass opacity, *ESR* erythrocyte sedimentation rate, *MCV* mean corpuscular erythrocyte volume, *MCH* mean corpuscular hemoglobin, *MCHC* mean corpuscular-hemoglobin concentration, *RDW.SD* red blood cell distribution width-standard deviation, *RDW.CV* red blood cell distribution width-variation coefficient, *TBIL* total bilirubin, *DBIL* direct bilirubin, *IBIL* indirect bilirubin, *ALT* alanine transaminase, *AST* aspartate transaminase, *ALP* alkaline phosphatase, *GGT* gamma-glutamyl transpeptidase, *LDH* lactate dehydrogenase, *HBDH* hydroxybutyrate-dehydrogenase, *HDL* high density lipoprotein, *LDL* low density lipoprotein, *CRP* C-reactive protein.

**Supplementary Table S2.** Characteristics of tuberculosis patients after 6-month treatment in the in-house cohort-1.

| **Characteristics** | **Anti-TB treatment** **success** **^*^ (n=3)** | **Anti-TB treatment failure ^*^ (n=2)** | **P** |
| --- | --- | --- | --- |
| **Demographic characteristics, *mean±standard deviation or n*** | | | |
| Age, years | 32.00±3.00 | 30.00±0.00 | 0.326 |
| Sex (Female/Male) | 2/1 | 0/2 | 0.400 |
| TB history (Yes/No) | 0/3 | 0/2 | - |
| TB contact history (Yes/No) | 1/2 | 1/1 | 1.000 |
| **Clinical symptoms, *n (%)*** | | | |
| Cough | 2 (66.67) | 1 (50.00) | 0.400 |
| Expectoration | 2 (66.67) | 0 (0.00) | 0.400 |
| Weight loss | 1 (33.33) | 1 (50.00) | 1.000 |
| Chest tightness | 1 (33.33) | 1 (50.00) | 1.000 |
| Insomnia | 0 (0.00) | 1 (50.00) | 0.400 |
| Hemoptysis | 1 (33.33) | 0 (0.00) | 1.000 |
| **Imaging characteristics, *n (%)*** | | | |
| Pulmonary GGO | 1 (33.33) | 0 (0.00) | 1.000 |
| Pulmonary cavity | 1 (33.33) | 0 (0.00) | 1.000 |
| Pulmonary nodule | 1 (33.33) | 1 (50.00) | 1.000 |
| Pulmonary patchy shadow | 2 (66.67) | 1 (50.00) | 1.000 |
| Pulmonary calcification | 1 (33.33) | 0 (0.00) | 1.000 |
| **TB-related detections, *n (%)*** | | | |
| TB-DNA | 0 (0.00) | - | - |
| Smear | 0 (0.00) | - | - |
| Culture | - | - | - |
| **Laboratory examinations,** ***mean±standard deviation or*** ***median (percent_25_- percent_75_)*** | | | |
| ***Whole blood-based detections*** | | | |
| ESR, mm/h | - | 13.50±10.61 | - |

**Continued**

| **Characteristics** | **Anti-TB Treatment success**  **(n=3)** | **Anti-TB Treatment failure**  **(n=2)** | **P** |
| --- | --- | --- | --- |
| Hemoglobin, g/L | 159.67±8.51 | 133.00±8.49 | 0.041 |
| Hematocrit, L/L | 0.47±0.02 | 0.39±0.01 | 0.017 |
| MCV, fL | 91.77±1.35 | 97.75±5.44 | 0.356 |
| MCH, pg | 31.23±0.65 | 33.40±1.13 | 0.067 |
| MCHC, g/L | 340.33±11.02 | 342.50±7.78 | 0.829 |
| RDW.SD, fL | 41.33±0.57 | 44.60±1.56 | 0.183 |
| RDW.CV, % | 12.23±0.21 | 12.45±0.35 | 0.437 |
| Platelets, 10^8^/L | 190±37.07 | 184±4.24 | 0.834 |
| White blood cell, 10^9^/L | 5.56 (5.46- -) | 6.26 (5.43- -) | 0.564 |
| Neutrophil, 10^9^/L | 2.97±0.06 | 3.02±0.25 | 0.817 |
| Lymphocyte, 10^9^/L | 2.13 (1.89- -) | 2.66 (2.14- -) | 0.454 |
| Monocyte, 10^9^/L | 0.42±0.14 | 0.41±0.13 | 0.921 |
| Eosinophil, 10^9^/L | 0.10±0.03 | 0.12±0.05 | 0.692 |
| Basophil, 10^9^/L | 0.03±0.02 | 0.05±0.00 | 0.133 |
| Neutrophil, % | 53.17±2.35 | 48.75±5.02 | 0.258 |
| Lymphocyte, % | 37.00±2.31 | 42.15±3.89 | 0.150 |
| Monocyte, % | 7.57±2.30 | 6.50±0.85 | 0.590 |
| Basophil, % | 0.47±0.25 | 0.80±0.14 | 0.197 |
| ***Serum-based detections*** | | | |
| TBIL, µmol/L | 17.63±5.30 | 6.30±2.83 | 0.075 |
| DBIL, µmol/L | 7.37±3.56 | 3.15±0.21 | 0.210 |
| IBIL, µmol/L | 10.27±1.99 | 3.15±3.04 | 0.047 |
| ALT, IU/L | 16.91±10.30 | 12.35±3.32 | 0.603 |
| AST, IU/L | 24.00 (22.00- -) | 20.45 (19.00- -) | 0.335 |
| AST/ALT | 1.26 (0.85- -) | 1.69 (1.49- -) | 0.648 |
| ALP, IU/L | 57.33±20.03 | 44.50±53.03 | 0.713 |

**Continued**

| **Characteristics** | **Anti-TB Treatment success**  **(n=3)** | **Anti-TB Treatment failure**  **(n=2)** | **P** |
| --- | --- | --- | --- |
| GGT, IU/L | 14.33±7.64 | 25.00±9.89 | 0.261 |
| Creatine kinase, IU/L | 127.50±30.41 | 88.00±7.07 | 0.215 |
| LDH, IU/L | 200.50±12.02 | 186.00±22.63 | 0.507 |
| HBDH, IU/L | 170.50±21.92 | 152.00±24.04 | 0.506 |
| Total Protein, g/L | 74.47±5.43 | 76.30±3.96 | 0.714 |
| Albumin, g/L | 49.17±1.62 | 47.35±4.03 | 0.638 |
| Globulin, g/L | 27.50 (18.20- -) | 28.95 (28.90- -) | 0.493 |
| Albumin/Globulin | 2.05±0.62 | 1.63±0.14 | 0.444 |
| Glucose, mmol/L | 4.91±0.44 | 4.77±0.23 | 0.727 |
| Urea, mmol/L | 4.40±0.87 | 5.35±0.35 | 0.255 |
| Creatinine, mmol/L | 70.33±19.43 | 62.00±7.07 | 0.616 |
| Cystatin C, mg/L | 0.90±0.33 | 0.71±0.12 | 0.501 |
| Triglycerides, mmol/L | 0.63±0.23 | 0.72±0.18 | 0.705 |
| HDL, mmol/L | 1.76±0.10 | 1.95±0.32 | 0.502 |
| LDL, mmol/L | 1.86±0.40 | 2.38±0.23 | 0.248 |
| CRP, mmol/L | - | - | - |

* Patients received the standard anti-tuberculosis therapy (2 months of isoniazid, rifampin, pyrazinamide and ethambutol, followed by 4 months of isoniazid and rifampin). *TB* tuberculosis, *GGO* ground-glass opacity, *ESR* erythrocyte sedimentation rate, *MCV* mean corpuscular erythrocyte volume, *MCH* mean corpuscular hemoglobin, *MCHC* mean corpuscular-hemoglobin concentration, *RDW.SD* red blood cell distribution width-standard deviation, *RDW.CV* red blood cell distribution width-variation coefficient, *TBIL* total bilirubin, *DBIL* direct bilirubin, *IBIL* indirect bilirubin, *ALT* alanine transaminase, *AST* aspartate transaminase, *ALP* alkaline phosphatase, *GGT* gamma-glutamyl transpeptidase, *LDH* lactate dehydrogenase, *HBDH* hydroxybutyrate-dehydrogenase, *HDL* high density lipoprotein, *LDL* low density lipoprotein, *CRP* C-reactive protein.

**Supplementary Table S3.** The statistics of Oxford Nanopore Technologies sequencing.

| **Sample** | **Total bases (G)** | **Read number** | **Mean length of read** | **N50 of read length** | **Mean quality of reads** |
| --- | --- | --- | --- | --- | --- |
| TB1 | 10.65 | 7,023,177 | 1,515 | 1,952 | 9.8 |
| TB2 | 9.64 | 6,494,303 | 1,484 | 1,931 | 9.7 |
| TB3 | 9.82 | 6,287,899 | 1,562 | 1,991 | 9.8 |
| TB4 | 9.45 | 5,729,158 | 1,649 | 2,176 | 10.2 |
| TB5 | 10.82 | 7,033,625 | 1,537 | 2,011 | 10.3 |
| HC1 | 9.88 | 6,951,090 | 1,421 | 1,863 | 9.8 |
| HC2 | 13.25 | 8,547,462 | 1,549 | 2,044 | 9.9 |
| HC3 | 10.29 | 6,794,689 | 1,514 | 1,966 | 9.8 |
| HC4 | 9.47 | 6,034,223 | 1,568 | 2,084 | 10.2 |
| HC5 | 10.48 | 6,840,753 | 1,531 | 1,978 | 10.3 |
| TB-F1 | 10.67 | 7,127,698 | 1,496 | 1,984 | 9.8 |
| TB-F2 | 11.38 | 8,164,083 | 1,393 | 1,876 | 10.3 |
| TB-S1 | 11.26 | 7,163,764 | 1,571 | 2,040 | 9.9 |
| TB-S2 | 11.80 | 7,726,777 | 1,527 | 2,059 | 10.4 |
| TB-S3 | 9.90 | 7,692,575 | 1,287 | 1,666 | 10.3 |

*TB* tuberculosis, *HC* healthy control, *TB-F* anti-tuberculosis treatment failure, *TB-S* anti-tuberculosis treatment success.

**Supplementary Table S4.** The characteristics of the in-house cohort-2.

| **Characteristics** | **Total (n=2,274)** | **Group** | | |
| --- | --- | --- | --- | --- |
|  |  | **Healthy controls (n=1,238)** | **Tuberculosis patients (n=1,036)** | **P** |
| **Demographic characteristics, *median (percent_25_- percent_75_) or n*** | | | | |
| Age, years | 39.50 (28.00-52.00) | 42.00 (32.00-52.00) | 35.00 (23.00-51.00) | <0.001 |
| Sex (Female/Male) | 1020/1254 | 565/673 | 455/581 | 0.674 |
| BMI | - | - | 20.03 (17.97-22.49) | - |
| Ethnicity (Han/Tibet/Yi/Others) | 1424/644/197/9 | 875/276/87/0 | 549/368/110/9 | <0.001 |
| **Personal history and complications, *n (%)*** | | | | |
| Smoking history | - | - | 440 (42.47) | - |
| Drinking history | - | - | 443 (42.76) | - |
| Tuberculosis-contact history | 304 (13.37) | 0 (0.00) | 304 (29.34) | <0.001 |
| Pervious tuberculosis history | 151 (6.64) | 0 (0.00) | 151 (14.58) | <0.001 |
| Syphilis | 164 (7.21) | 0 (0.00) | 164 (15.83) | <0.001 |
| AIDS | 254 (11.17) | 0 (0.00) | 254 (24.52) | <0.001 |
| Parasite | 360 (15.83) | 0 (0.00) | 360 (34.75) | <0.001 |
| Unknown infectious disease | 435 (19.13) | 0 (0.00) | 435 (41.99) | <0.001 |
| Hepatitis | 302 (13.28) | 0 (0.00) | 302 (29.15) | <0.001 |
| Other hepatic disease | 358 (15.74) | 0 (0.00) | 358 (34.56) | <0.001 |
| Psychological disorder | 318 (13.98) | 0 (0.00) | 318 (30.69) | <0.001 |
| Sleep disorders | 334 (14.69) | 0 (0.00) | 334 (32.24) | <0.001 |
| Cerebral disease | 317 (13.94) | 0 (0.00) | 317 (30.60) | <0.001 |
| Cardiac disease | 370 (16.27) | 0 (0.00) | 370 (35.71) | <0.001 |
| Renal dysfunction | 344 (15.13) | 0 (0.00) | 344 (33.20) | <0.001 |
| Cancer | 327 (14.38) | 0 (0.00) | 327 (31.56) | <0.001 |
| Diabetes mellitus | 341 (15.00) | 0 (0.00) | 341 (32.92) | <0.001 |
| Electrolytes disorder | 385 (16.93) | 0 (0.00) | 385 (37.16) | <0.001 |
| Anemia | 361 (15.88) | 0 (0.00) | 361 (34.85) | <0.001 |

**Continued**

| **Characteristics** | **Total (n=2,274)** | **Group** | | |
| --- | --- | --- | --- | --- |
|  |  | **Healthy controls (n=1,238)** | **Tuberculosis patients (n=1,036)** | **P** |
| Hypertension | 359 (15.79) | 0 (0.00) | 359 (34.65) | <0.001 |
| Hyperlipemia | 337 (14.82) | 0 (0.00) | 337 (32.53) | <0.001 |
| PH | 371 (16.31) | 0 (0.00) | 371 (35.81) | <0.001 |
| Hypoproteinemia | 405 (17.81) | 0 (0.00) | 405 (39.09) | <0.001 |
| **Drugs on other disease, *n (%).*** | | | | |
| Hypotensive drugs | 225 (9.89) | 0 (0.00) | 225 (21.72) | <0.001 |
| Hypoglycemic drugs | 212 (9.32) | 0 (0.00) | 212 (20.46) | <0.001 |
| Prednisone | 268 (11.79) | 0 (0.00) | 268 (25.87) | <0.001 |
| Hepatic protector | 330 (14.51) | 0 (0.00) | 330 (31.85) | <0.001 |
| **Clinical symptoms, *n (%)*** | | | | |
| Cough | 496 (21.81) | 0 (0.00) | 496 (47.88) | <0.001 |
| Chest pain | 232 (10.20) | 0 (0.00) | 232 (22.39) | <0.001 |
| Fatigue | 330 (14.51) | 0 (0.00) | 330 (31.85) | <0.001 |
| Fever | 349 (15.35) | 0 (0.00) | 349 (33.69) | <0.001 |
| Hemoptysis | 330 (14.51) | 0 (0.00) | 330 (31.85) | <0.001 |
| Night sweat | 362 (15.92) | 0 (0.00) | 362 (34.94) | <0.001 |
| **Imaging characteristics, *n (%)*** | | | | |
| Pulmonary cavity | 225 (9.89) | 0 (0.00) | 225 (21.72) | <0.001 |
| Pulmonary calcification | 367 (16.14) | 0 (0.00) | 367 (35.42) | <0.001 |
| Miliary mottling | 40 (1.76) | 0 (0.00) | 40 (3.86) | <0.001 |
| Pulmonary nodule | 364 (16.01) | 0 (0.00) | 364 (35.14) | <0.001 |
| Pneumonedema | 380 (16.71) | 0 (0.00) | 380 (36.68) | <0.001 |
| Lymphadenopathy | 376 (16.53) | 0 (0.00) | 376 (36.29) | <0.001 |
| **Microbiological diagnosis, *n (%)*** | | | | |
| Smear | 166 (7.30) | 0 (0.00) | 166 (16.02) | <0.001 |

**Continued**

| **Characteristics** | **Total (n=2,274)** | **Group** | | |
| --- | --- | --- | --- | --- |
|  |  | **Healthy controls (n=1,238)** | **Tuberculosis patients (n=1,036)** | **P** |
| IGRA | 602 (26.47) | 0 (0.00) | 602 (58.11) | <0.001 |
| Culture | 349 (15.35) | 0 (0.00) | 349 (33.69) | <0.001 |
| TB-DNA | 484 (21.28) | 0 (0.00) | 484 (46.72) | <0.001 |
| Xpert | 507 (22.30) | 0 (0.00) | 507 (48.94) | <0.001 |
| Tuberculosis-antibody | 487 (21.42) | 0 (0.00) | 487 (47.01) | <0.001 |
| **Laboratory examinations, *median (percent_25_- percent_75_)*** | | | | |
| ***Whole blood-based detections*** | | | | |
| ESR, mm/h | - | - | 24.00 (8.00-50.00) | - |
| Hemoglobin, g/L | 134.00 (104.00-151.00) | 133.00 (90.00-152.00) | 135.00 (120.00-150.00) | <0.001 |
| Hematocrit, L/L | 0.46 (0.42-0.55) | 0.45 (0.42-0.48) | 32.25 (0.42-42.00) | <0.001 |
| MCV, fL | 88.50 (76.30-93.60) | 89.40 (65.90-94.30) | 87.80 (82.68-92.50) | 0.012 |
| MCH, pg | 29.20 (26.20-30.90) | 29.30 (25.40-31.00) | 29.10 (26.90-30.70) | 0.211 |
| MCHC, g/L | 331.00 (322.00-345.00) | 333.00 (324.00-357.00) | 329.00 (320.00-339.00) | <0.001 |
| RDW.SD, fL | 38.10 (13.50-43.90) | 39.50 (13.40-43.90) | 19.65 (13.60-43.80) | 0.557 |
| RDW.CV, % | 39.40 (13.30-44.80) | 40.60 (13.20-45.20) | 37.40 (13.80-43.40) | 0.098 |
| Platelets, 10^8^/L | 236.00 (187.00-312.00) | 231.00 (187.00-312.00) | 243.00 (189.00-323.00) | 0.010 |
| White blood cell, 10^9^/L | 5.89 (4.97-7.06) | 5.83 (5.03-6.75) | 6.08 (4.86-7.70) | <0.001 |
| Neutrophil, 10^9^/L | 55.12 (44.92-63.00) | 55.61 (50.78-61.28) | 52.43 (4.78-66.63) | <0.001 |
| Lymphocyte, 10^9^/L | 28.06 (14.02-35.11) | 32.96 (28.22-37.91) | 11.20 (1.43-25.40) | <0.001 |
| Monocyte, 10^9^/L | 6.60 (4.71-8.14) | 7.04 (6.09-8.38) | 5.20 (0.52-7.80) | <0.001 |
| Eosinophil, 10^9^/L | 1.70 (0.52-3.06) | 2.30 (1.42-3.60) | 0.50 (0.10-2.00) | <0.001 |
| Basophil, 10^9^/L | 0.50 (0.20-0.77) | 0.64 (0.47-0.85) | 0.20 (0.03-0.50) | <0.001 |
| Neutrophil, % | 3.72 (2.81-5.96) | 3.16 (2.60-3.95) | 6.76 (3.63-67.70) | <0.001 |
| Lymphocyte, % | 2.01 (1.48-2.87) | 1.98 (1.63-2.38) | 2.06 (1.26-17.93) | <0.001 |
| Monocyte, % | 0.45 (0.34-0.73) | 0.39 (0.32-0.49) | 0.73 (0.42-6.80) | <0.001 |

**Continued**

| **Characteristics** | **Total (n=2,274)** | **Group** | | |
| --- | --- | --- | --- | --- |
|  |  | **Healthy controls (n=1,238)** | **Tuberculosis patients (n=1,036)** | **P** |
| Eosinophil, % | 0.17 (0.08-0.33) | 0.15 (0.08-0.24) | 0.20 (0.09-1.20) | <0.001 |
| Basophil, % | 0.04 (0.02-0.07) | 0.04 (0.02-0.05) | 0.05 (0.02-0.30) | <0.001 |
| ***Serum-based detections*** | | | | |
| TBIL, mol/L | 11.30 (8.13-15.00) | 12.40 (10.00-15.80) | 9.20 (6.20-13.80) | <0.001 |
| DBIL, µmol/L | 3.10 (2.20-4.40) | 2.90 (2.20-3.90) | 3.50 (2.30-5.53) | <0.001 |
| IBIL, µmol/L | 7.90 (5.20-10.80) | 9.30 (7.30-12.30) | 5.50 (3.50-8.40) | <0.001 |
| ALT, IU/L | 21.50 (13.00-67.00) | 32.00 (16.00-118.00) | 16.00 (11.00-28.00) | <0.001 |
| AST, IU/L | 23.00 (18.00-45.00) | 27.00 (19.00-70.00) | 21.00 (16.00-27.00) | <0.001 |
| AST/ALT | 1.04 (0.67-1. 4550) | 0.79 (0.55-1.21) | 1.29 (0.92-1.73) | <0.001 |
| ALP, IU/L | 71.50 (57.00-94.00) | 60.00 (50.00-75.75) | 90.00 (69.00-115.00) | <0.001 |
| GGT, IU/L | 34.00 (17.00-105.00) | 39.00 (17.00-108.00) | 31.50 (17.00-64.25) | <0.001 |
| Creatine kinase, IU/L | 64.00 (39.00-97.00) | 73.50 (39.00-109.00) | 56.00 (35.00-82.00) | <0.001 |
| LDH, IU/L | 189.00 (164.00-221.00) | 195.00 (172.00-221.00) | 180.50 (149.75-220.00) | <0.001 |
| HBDH, IU/L | 144.00 (124.00-169.00) | 147.00 (130.00-167.00) | 137.00 (115.00-170.50) | <0.001 |
| Total Protein, g/L | 72.50 (69.03-76.20) | 73.30 (70.60-76.40) | 70.80 (65.00-75.50) | <0.001 |
| Albumin, g/L | 45.80 (40.90-48.50) | 47.50 (45.70-49.40) | 40.05 (34.98-44.90) | <0.001 |
| Globulin, g/L | 27.10 (24.40-30.60) | 25.90 (23.90-28.30) | 29.80 (25.70-34.90) | <0.001 |
| Albumin/Globulin | 1.83 (1. 6544-2.02) | 0.79 (0.55-1.21) | 1.29 (0..9203-1.73) | <0.001 |
| Glucose, mmol/L | 4.97 (4.63-5.60) | 5.14 (4.67-6.43) | 4.83 (4.46-5.31) | <0.001 |
| Urea, mmol/L | 4.70 (3.80-5.90) | 5.00 (4.20-6.00) | 4.30 (3.40-5.79) | <0.001 |
| Creatinine, mmol/L | 73.75 (59.20-91.00) | 87.00 (72.00-99.00) | 61.00 (51.00-73.00) | <0.001 |
| Cystatin C, mg/L | 0.90 (0.77-1.20) | 0.89 (0.78-1.19) | 0.91 (0.74-1.26) | 0.222 |
| Uric acid, mmol/L | 385.00 (294.00-488.00) | 401.00 (317.00-518.00) | 355.00 (267.00-472.25) | <0.001 |
| Triglycerides, mmol/L | 1.08 (0.77-1.60) | 1.22 (0.78-1.90) | 1.00 (0.76-1.33) | <0.001 |
| Cholesterol, mmol/L | 4.63 (3.81-5.62) | 5.33 (4.52-6.14) | 3.90 (3.26-4.63) | <0.001 |

**Continued**

| **Characteristics** | **Total (n=2,274)** | **Group** | | |
| --- | --- | --- | --- | --- |
|  |  | **Healthy controls**  **(n=1,238)** | **Tuberculosis patients (n=1,036)** | **P** |
| HDL, mmol/L | 1.15 (0.91-1.43) | 1.17 (0.96-1.45) | 1.12 (0.86-1.39) | <0.001 |
| LDL, mmol/L | 2.79 (2.14-3.95) | 3.59 (2.65-4.58) | 2.25 (1.81-2.80) | <0.001 |
| CRP, mg/L | 2.60 (1.40-11.73) | 1.84 (1.37-2.70) | 13.96 (2.60-40.90) | <0.001 |
| **Anti-tuberculosis drugs*, n (%)*** | | | | |
| Isoniazid | 656 (28.85) | 0 (0.00) | 656 (63.32) | <0.001 |
| Rifampicin | 533 (23.44) | 0 (0.00) | 533 (51.45) | <0.001 |
| Pyrazinamide | 613 (26.96) | 0 (0.00) | 613 (59.17) | <0.001 |
| Ethambutol | 681 (29.95) | 0 (0.00) | 681 (65.73) | <0.001 |
| Ofloxacin | 251 (11.04) | 0 (0.00) | 251 (24.23) | <0.001 |
| Amikacin | 185 (8.14) | 0 (0.00) | 185 (17.86) | <0.001 |
| Vitamin B6 | 169 (7.43) | 0 (0.00) | 169 (16.31) | <0.001 |
| Moxifloxacin | 199 (8.75) | 0 (0.00) | 199 (19.21) | <0.001 |
| Rifapentine | 231 (10.16) | 0 (0.00) | 231 (22.30) | <0.001 |

*BMI* body mass index, *AIDS* acquired immune deficiency syndrome, *PH* pulmonary artery hypertension, *IGRA* interferon-γ release assay, *ESR* erythrocyte sedimentation rate, *MCV* mean corpuscular erythrocyte volume, *MCH* mean corpuscular hemoglobin, *MCHC* mean corpuscular-hemoglobin concentration, *RDW.SD* red blood cell distribution width-standard deviation, *RDW.CV* red blood cell distribution width-variation coefficient, *TBIL* total bilirubin, *DBIL* direct bilirubin, *IBIL* indirect bilirubin, *ALT* alanine transaminase, *AST* aspartate transaminase, *ALP* alkaline phosphatase, *GGT* gamma-glutamyl transpeptidase, *LDH* lactate dehydrogenase, *HBDH* hydroxybutyrate-dehydrogenase, *HDL* high density lipoprotein, *LDL* low density lipoprotein, *CRP* C-reactive protein.

**Supplementary Table S5.** The information of included public datasets.

| **ID** | **Platform** | **Sex**  **(male:female)** | **Age**  **(years, mean±SD)** | **HIV status** | **TB diagnosis** | **Treatment** | **Group** | **Sample type** |
| --- | --- | --- | --- | --- | --- | --- | --- | --- |
| GSE79362 | GPL11154 | 105:250 | 15.69±0.08 | NA | Sputum smear or culture | / | Progression: 110 samples;  Non-progression: 245 samples | Whole blood |
| GSE94438 | GPL11154 | 177:251 | 27.72±0.59 | 0 | Clinical diagnosis | / | Progression: 101 samples;  Non-progression: 327 samples | Whole blood |
| GSE107994 and GSE107993 | GPL20301 | 7:2 | 46.33±5.00 | 0 | Culture | / | Progression: 3 samples;  Non-progression: 6 samples | Whole blood |
| GSE84076 | [GPL16791](https://www.ncbi.nlm.nih.gov/geo/query/acc.cgi?acc=GPL16791) | NA | NA | NA | Sputum smear and/or culture | NA | Treatment: 2 samples;  Pre-treatment: 6 samples | Whole blood |
| GSE89403 | GPL11154 | NA | NA | 0 | Sputum culture | Standard treatment | Treatment: 564 samples;  Pre-treatment: 182 samples | Whole blood |
| GSE122485 | GPL15520 | 11:0 | 31.91±8.86 | 0 | Smear and culture | Standard treatment | Treatment: 7 samples;  Pre-treatment: 4 samples | Whole blood |

*SD* standard deviation, *TB* tuberculosis, *NA* non-available.

**Supplementary Table S6.** The results of subgroup analysis for the tuberculosis-progression cohort.

|  | | **RPS20 -exon1-AP** | | | **KIF13B-exon4-SE** | | | **UBE2B-exon7-SE** | | |
| --- | --- | --- | --- | --- | --- | --- | --- | --- | --- | --- |
|  |  | **Non-progression** | **Progression** | **P** | **Non-progression** | **Progression** | **P** | **Non-progression** | **Progression** | **P** |
| Age (years) | <20 | 0.62 (0.01) | 0.58 (0.02) | 0.060 | 1.00 (0.00) | 1.00 (0.00) | 0.272 | 0.10 (0.00) | 0.10 (0.00) | 0.158 |
|  | 20-40 | 0.83 (0.01) | 0.81 (0.03) | 0.472 | 1.00 (0.00) | 1.00 (0.00) | 0.606 | 0.08 (0.00) | 0.09 (0.00) | 0.191 |
|  | 40-60 | 0.77 (0.03) | 0.78 (0.06) | 0.839 | 1.00 (0.00) | 1.00 (0.00) | 0.562 | 0.09 (0.00) | 0.08 (0.01) | 0.713 |
|  | ≥60 | 0.73 (0.24) | - | - | 1.00 (0.00) | - | - | 0.06 (0.04) | - | - |
|  | P | - | - | - | - | - | - | - | - | - |
| Sex | Female | 0.69 (0.01) | 0.65 (0.02) | 0.072 | 1.00 (0.00) | 1.00 (0.00) | 0.698 | 0.09 (0.00) | 0.09 (0.00) | 0.734 |
|  | Male | 0.70 (0.02) | 0.70 (0.03) | 0.881 | 1.00 (0.00) | 1.00 (0.00) | 0.822 | 0.09 (0.00) | 0.09 (0.00) | 0.551 |
|  | P | 0.476 | 0.178 | - | 0.065 | 0.327 | - | 0.279 | 0.265 | - |
| TB history | No | 0.55 (0.01) | 0.54 (0.02) | 0.740 | 1.00 (0.00) | 1.00 (0.00) | 0.489 | 0.11 (0.00) | 0.10 (0.00) | 0.004 |
|  | Yes | 0.52 (0.03) | 0.43 (0.03) | 0.171 | 1.00 (0.00) | 1.00 (0.00) | 0.741 | 0.10 (0.01) | 0.09 (0.01) | 0.446 |
|  | P | 0.378 | 0.127 | - | 0.419 | 0.766 | - | 0.152 | 0.470 | - |
| Time to exposure (months) | 0 | 0.81 (0.01) | 0.81 (0.02) | 0.915 | 1.00 (0.00) | 1.00 (0.00) | 0.082 | 0.08 (0.00) | 0.09 (0.00) | 0.160 |
|  | 6 | 0.86 (0.02) | 0.82 (0.04) | 0.309 | 1.00 (0.00) | 1.00 (0.00) | 0.894 | 0.09 (0.00) | 0.09 (0.00) | 0.966 |
|  | 18 | 0.81 (0.02) | 0.89 (0.04) | 0.076 | 1.00 (0.00) | 1.00 (0.00) | 0.346 | 0.08 (0.00) | 0.09 (0.01) | 0.417 |
|  | P | - | - | - | - | - | - | - | - | - |

**Continued**

|  | | **RPS20-exon1-AP** | | | **KIF13B-exon4-SE** | | | **UBE2B-exon7-SE** | | |
| --- | --- | --- | --- | --- | --- | --- | --- | --- | --- | --- |
|  |  | **Non-progression** | **Progression** | **P** | **Non-progression** | **Progression** | **P** | **Non-progression** | **Progression** | **P** |
| Time to TB (months) | NA | 0.82 (0.01) | - | - | 1.00 (0.00) | - | - | 0.08 (0.00) | - | - |
|  | < 6 | - | 0.75 (0.04) | 0.055 ^*^ | - | 1.00 (0.00) | 0.503 ^*^ | - | 0.09 (0.01) | 0.623 ^*^ |
|  | 6-12 | - | 0.85 (0.03) | 0.447 ^*^ | - | 1.00 (0.00) | 0.075 ^*^ | - | 0.09 (0.00) | 0.070 ^*^ |
|  | 12-18 | - | 0.82 (0.03) | 0.948 ^*^ | - | 1.00 (0.00) | 0.827 ^*^ | - | 0.09 (0.01) | 0.698 ^*^ |
|  | 18-24 | - | 0.85 (0.03) | 0.288 ^*^ | - | 1.00 (0.00) | 0.771 ^*^ | - | 0.09 (0.00) | 0.587 ^*^ |
|  | *P* | *-* | *-* | *-* | *-* | *-* | *-* | *-* | *-* | *-* |

* P values were used to evaluate the difference between the non-progressed participants and tuberculosis patients with different progression time. *RPS20* Ribosomal Protein S20, *AP* alternative promoter, *KIF13B* Kinesin Family Member 13B, *SE* skipping exon, *UBE2B* Ubiquitin Conjugating Enzyme E2 B, *TB* tuberculosis, *NA* non-available.

**Supplementary Table S7.** The results of subgroup analysis for the tuberculosis-treatment cohort.

|  | | **RPS20 -exon1-AP** | | | **KIF13B-exon4-SE** | | | **UBE2B-exon7-SE** | | |
| --- | --- | --- | --- | --- | --- | --- | --- | --- | --- | --- |
|  |  | **Pre-treatment** | **Treatment** | **P** | **Pre-treatment** | **Treatment** | **P** | **Pre-treatment** | **Treatment** | **P** |
| Treatment duration | NA | 0.53 (0.02) | - | - | 1.00 (0.00) | - | - | 0.07 (0.00) | - | - |
|  | 1 week | - | 0.55 (0.01) | 0.513^*^ | - | 1.00 (0.00) | 0.868^*^ | - | 0.06 (0.00) | 0.093^*^ |
|  | 1 month | - | 0.55 (0.01) | 0.313^*^ | - | 1.00 (0.00) | 0.171^*^ | - | 0.06 (0.00) | 0.156^*^ |
|  | 6 months | - | 0.54 (0.01) | 0.717^*^ | - | 1.00 (0.00) | 0.850^*^ | - | 0.06 (0.00) | 0.412^*^ |
|  | 12 months | - | 0.00 (0.00) | <0.001^*^ | - | 1.00 (0.00) | 0.290^*^ | - | 0.13 (0.07) | 0.032^*^ |
| Treatment outcome | Not cure | 0.53 (0.03) | | - | 1.00 (0.00) | | - | 0.06 (0.00) | | - |
|  | Possible cure | 0.55 (0.03) | | 0.490^**^ | 1.00 (0.00) | | 0.288^**^ | 0.06 (0.00) | | 0.393^**^ |
|  | Probable cure | 0.55 (0.02) | | 0.513^**^ | 1.00 (0.00) | | 0.767^**^ | 0.07 (0.00) | | 0.107^**^ |
|  | Definite cure | 0.55 (0.01) | | 0.469^**^ | 1.00 (0.00) | | 0.725^**^ | 0.06 (0.00) | | 0.344^**^ |
|  | Unevaluable outcome | 0.61 (0.07) | | 0.205^**^ | 1.00 (0.00) | | 0.882^**^ | 0.04 (0.01) | | 0.006^**^ |

* P values were used to evaluate the difference between tuberculosis patients without treatment and with different treatment duration. ** P values were calculated by comparing patients in the possible cure, probable cure, definite cure or unevaluable outcome groups with those in the no cure group. *RPS20* Ribosomal Protein S20, *AP* alternative promoter, *KIF13B* Kinesin Family Member 13B, *SE* skipping exon, *UBE2B* Ubiquitin Conjugating Enzyme E2 B, *NA* non-available.

**Supplementary Table S8.** The results of the variable selection.

| **Variable ^*^** | **Odd ratio** | **95% confidence interval** | **P** |
| --- | --- | --- | --- |
| Hematocrit | 38.407 | 16.409-89.895 | 4.16e-17 |
| Ethnicity | 1.826 | 1.578-2.112 | 6.06e-16 |
| White blood cells | 1.169 | 1.115-1.225 | 9.72e-11 |
| Hemoglobin | 1.012 | 1.008-1.015 | 5.37e-12 |
| Platelet | 1.002 | 1.001-1.003 | 3.52e-4 |
| RDW.SD | 0.99 | 0.984-0.995 | 4.73e-4 |
| Age | 0.979 | 0.973-0.985 | 2.46e-11 |
| Neutrophil percent | 0.966 | 0.961-0.97 | 4.69e-48 |
| Lymphocyte percent | 0.854 | 0.842-0.867 | 3.14e-101 |
| Monocyte percent | 0.763 | 0.738-0.789 | 8.79e-56 |
| Eosinophil percent | 0.67 | 0.629-0.714 | 5.42e-35 |
| Basophil percent | 0.044 | 0.031-0.062 | 1.11e-69 |
| Sex | 1.162 | 0.965-1.400 | 0.11 |
| RDW.CV | 0.996 | 0.991-1.002 | 0.22 |

* Original clinical indicators were included, while indicators that were calculated based on current items, such as lymphocytes, were not. *RDW.SD* red blood cell distribution width-standard deviation, *RDW.CV* red blood cell distribution width-variation coefficient.

**Supplementary Table S9.** The variance inflation factor of imputed variables.

|  | **Training set** | **Test set** |
| --- | --- | --- |
| Hematocrit | 1.037 | 1.018 |
| RPS20-exon1-AP | 1.074 | 1.058 |
| KIF13B -exon4-SE | 1.048 | 1.023 |
| UBE2B-exon7-SE | 1.080 | 1.065 |

*RPS20* Ribosomal Protein S20, *AP* alternative promoter, *KIF13B* Kinesin Family Member 13B, *SE* skipping exon, *UBE2B* Ubiquitin Conjugating Enzyme E2 B.

**Supplementary Table S10.** The assessment of modeling according to TRIPOD.

| **Item** | **Development or validation** | **Checklist item** | **Result** |
| --- | --- | --- | --- |
| 1 | Development | Identify the study as developing and/or validating a multivariable prediction model, the target population, and the outcome to be predicted | Yes |
| 2 | Development | Provide a summary of objectives, study design, setting, participants, sample size, predictors, outcome, statistical analysis, results, and conclusions | Yes |
| 3a | Development | Explain the medical context (including whether diagnostic or prognostic) and rationale for developing or validating the multivariable prediction model, including references to existing models | Yes |
| 3b | Development | Specify the objectives, including whether the study describes the development or validation of the model, or both | Yes |
| 4a | Development | Describe the study design or source of data (for example, randomized trial, cohort, or registry data), separately for the development and validation data sets, if applicable | Yes |
| 4b | Development | Specify the key study dates, including start of accrual; end of accrual; and, if applicable, end of follow-up | Yes |
| 5a | Development | Specify key elements of the study setting (for example, primary care, secondary care, general population) including number and location of centres | Yes |
| 5b | Development | Describe eligibility criteria for participants | Yes |
| 5c | Development | Give details of treatments received, if relevant | Yes |
| 6a | Development | Clearly define the outcome that is predicted by the prediction model, including how and when assessed | Yes |
| 6b | Development | Report any actions to blind assessment of the outcome to be predicted | Yes |
| 7a | Development | Clearly define all predictors used in developing the multivariable prediction model, including how and when they were measured | Yes |
| 7b | Development | Report any actions to blind assessment of predictors for the outcome and other predictors | Yes |
| 8 | Development | Explain how the study size was arrived at | Yes |
| 9 | Development | Describe how missing data were handled (for example, complete-case analysis, single imputation, multiple imputation) with details of any imputation method | Yes |
| 10a | Development | Describe how predictors were handled in the analyses | Yes |

**Continued**

| **Item** | **Development or validation** | **Checklist item** | **Result** |
| --- | --- | --- | --- |
| 10b | Development | Specify type of model, all model-building procedures (including any predictor selection), and method for internal validation | Yes |
| 10c | Validation | For validation, describe how the predictions were calculated | Yes |
| 10d | Development | Specify all measures used to assess model performance and, if relevant, to compare multiple models | Yes |
| 10e | Development | Describe any model updating (for example, recalibration) arising from the validation, if done | Yes |
| 11 | Development | Provide details on how risk groups were created, if done | Yes |
| 12 | Validation | For validation, identify any differences from the development data in setting, eligibility criteria, outcome, and predictors | Yes |
| 13a | Development | Describe the flow of participants through the study, including the number of participants with and without the outcome and, if applicable, a summary of the follow-up time. A diagram may be helpful | Yes |
| 13b | Development | Describe the characteristics of the participants (basic demographics, clinical features, available predictors), including the number of participants with missing data for predictors and outcome | Yes |
| 13c | Validation | For validation, show a comparison with the development data of the distribution of important variables (demographics, predictors and outcome). | Yes |
| 14a | Development | Specify the number of participants and outcome events in each analysis | Yes |
| 14b | Development | If done, report the unadjusted association between each candidate predictor and outcome | Yes |
| 15a | Development | Present the full prediction model to allow predictions for individuals (that is, all regression coefficients, and model intercept or baseline survival at a given time point) | Yes |
| 15b | Development | Explain how to use the prediction model | Yes |
| 16 | Development | Report performance measures (with CIs) for the prediction model | Yes |
| 17 | Validation | If done, report the results from any model updating (that is, model specification, model performance) | Yes |

**Continued**

| **Item** | **Development or validation** | **Checklist item** | **Result** |
| --- | --- | --- | --- |
| 18 | Development | Discuss any limitations of the study (such as nonrepresentative sample, few events per predictor, missing data) | Yes |
| 19a | Validation | For validation, discuss the results with reference to performance in the development data, and any other validation data | Yes |
| 19b | Development | Give an overall interpretation of the results, considering objectives, limitations, results from similar studies, and other relevant evidence | Yes |
| 20 | Development | Discuss the potential clinical use of the model and implications for future research | Yes |
| 21 | Development | Provide information about the availability of supplementary resources, such as study protocol, Web calculator, and data sets | Yes |
| 22 | Development | Give the source of funding and the role of the funders for the present study | Yes |

**Supplementary Table S11.** The sequence of primers.

| **Experiment** | **Gene** | **Forward primer** | **Reverse primer** |
| --- | --- | --- | --- |
| qRT-PCR | GAPDH | CCTTCCGTGTCCCCACT | GCCTGCTTCACCACCTTC |
|  | S100A8-intron1-inclusion | CAGCTGTCTTTCAGAAGACCTGGTG | GACGTCGATGATAGAGTTCAAGGC |
|  | S100A8-intron1-exclusion | GCATGAATCCTCTGCGTACTGCC | GCCCCACCTGAAAAACAGAAC |
|  | RPS20-exon1-inclusion | CCATGGCTTTTAAGGATACCG | CGGCTTGTTAGGGTGATTCG |
|  | RPS20-exon1-exclusion | GGCGCTCCTGAAATGGCTT | CGGCTTGTTAGGGTGATTCG |
|  | KIF13B-exon4-inclusion | GATGCCCGGTGTTTGCTTATG | GGATGCTGGATTCTATTACCTG |
|  | KIF13B-exon4-exclusion | CAAATGTGTGGTGGATGTGGA | ATCATAAGCAAACACCTTCGG |
|  | UBE2B-exon7-inclusion | ACATCAATTCAGTCTCTGC | GTTTTCCTGATAAAGCTGTGCTG |
|  | UBE2B-exon7-exclusion | TCAGTGTCTTGCTCTGTTGC | TGGCTGGACTGTTAGGATTCG |
|  | SRSF1 | CAACGATTGCCGCATCTACG | TCGAACTCAACGAAGGCGAA |
| RIP-qPCR | UBE2B | GCTGGATGAACCGAATCCTA | TTCAACAATGGCCGAAACTC |

*qRT-PCR* quantificational real-time polymerase chain reaction, *GAPDH* Glyceraldehyde-3-Phosphate Dehydrogenase, *S100A8* S100 Calcium Binding Protein A8, *RPS20* Ribosomal Protein S20, *KIF13B* Kinesin Family Member 13B, *UBE2B* Ubiquitin Conjugating Enzyme E2 B, *SRSF1* serine/arginine-rich splicing factor 1, *RIP* RNA immunoprecipitation.
